# Intestinal *Bacteroides* modulates inflammation, systemic cytokines, and microbial ecology via propionate in a mouse model of cystic fibrosis

**DOI:** 10.1128/mbio.03144-23

**Published:** 2024-01-05

**Authors:** Courtney E. Price, Rebecca A. Valls, Alexis R. Ramsey, Nicole A. Loeven, Jane T. Jones, Kaitlyn E. Barrack, Joseph D. Schwartzman, Darlene B. Royce, Robert A. Cramer, Juliette C. Madan, Benjamin D. Ross, James Bliska, George A. O'Toole

**Affiliations:** 1 Department of Microbiology and Immunology, Geisel School of Medicine at Dartmouth, Hanover, USA; 2 Department of Pathology, Geisel School of Medicine at Dartmouth, Hanover, USA; 3 Department of Psychiatry, Geisel School of Medicine at Dartmouth, Hanove, USA; University of Washington School of Medicine, Seattle, Washington, USA

**Keywords:** cystic fibrosis, gut, SCFA, propionate, inflammation, probiotic

## Abstract

Persons with cystic fibrosis (CF), starting in early life, show intestinal microbiome dysbiosis characterized in part by a decreased relative abundance of the genus *Bacteroides. Bacteroides* is a major producer of the intestinal short chain fatty acid propionate. We demonstrate here that cystic fibrosis transmembrane conductance regulator-defective (CFTR−/−) Caco-2 intestinal epithelial cells are responsive to the anti-inflammatory effects of propionate. Furthermore, *Bacteroides* isolates inhibit the IL-1β-induced inflammatory response of CFTR−/− Caco-2 intestinal epithelial cells and do so in a propionate-dependent manner. The introduction of *Bacteroides-*supplemented stool from infants with cystic fibrosis into the gut of *Cftr^F508del^* mice results in higher propionate in the stool as well as the reduction in several systemic pro-inflammatory cytokines. *Bacteroides* supplementation also reduced the fecal relative abundance of *Escherichia coli*, indicating a potential interaction between these two microbes, consistent with previous clinical studies. For a *Bacteroides* propionate mutant in the mouse model, pro-inflammatory cytokine KC is higher in the airway and serum compared with the wild-type (WT) strain, with no significant difference in the absolute abundance of these two strains. Taken together, our data indicate the potential multiple roles of *Bacteroides*-derived propionate in the modulation of systemic and airway inflammation and mediating the intestinal ecology of infants and children with CF. The roles of *Bacteroides* and the propionate it produces may help explain the observed gut-lung axis in CF and could guide the development of probiotics to mitigate systemic and airway inflammation for persons with CF.

**IMPORTANCE:**

The composition of the gut microbiome in persons with CF is correlated with lung health outcomes, a phenomenon referred to as the gut-lung axis. Here, we demonstrate that the intestinal microbe *Bacteroides* decreases inflammation through the production of the short-chain fatty acid propionate. Supplementing the levels of *Bacteroides* in an animal model of CF is associated with reduced systemic inflammation and reduction in the relative abundance of the opportunistically pathogenic group *Escherichia*/*Shigella* in the gut. Taken together, these data demonstrate a key role for *Bacteroides* and microbially produced propionate in modulating inflammation, gut microbial ecology, and the gut-lung axis in cystic fibrosis. These data support the role of *Bacteroides* as a potential probiotic in CF.

## INTRODUCTION

Cystic fibrosis (CF) is a common heritable disease with multi-organ effects that ultimately reduce the quality of life and life expectancy for persons with CF (pwCF) (1). CF is caused by a defect in the cystic fibrosis transmembrane conductance regulator (CFTR), which leads to dysregulation of ion and water flow and an accumulation of thick, sticky mucus at mucosal surfaces (2). In the lung, this mucosal alteration leads to colonization and chronic infection with polymicrobial communities of opportunistic pathogens (3, 4). The physiologic effects of the CFTR mutation on the gut include risk of meconium ileus at birth, longer gastrointestinal transit times, and dysbiosis of the gut microbiota (5–8). Until relatively recently, CF microbiology research focused almost exclusively on the lung microbiota, as the majority of modern CF-related deaths are attributable to respiratory complications (1, 9, 10). However, interest in the CF gut microbiome and its influence on distal organ health has grown as the role of the gut microbiome in broader health outcomes has become more apparent (5, 11–15).

The human gut microbiome is comprised of diverse microorganisms that influence a broad range of host health outcomes, including normal immune development and function (16). Cross-talk between the gut and the lung has been referred to as the “gut-lung axis,” and this connection between the gut and the lung has been proposed to play a role in a number of airway diseases, including CF (17). Both infants and adults with CF have a dysbiotic gut microbiome (5, 7, 11, 13, 18–20), and there is a significant correlation between gut microbiota beta-diversity for infants and children with CF and the risk of airway exacerbation in this population (5, 14). Additionally, reduced microbial diversity in the gut microbiota correlates with earlier onset of the first clinical exacerbation in infants with CF (14). Studies from our group and others have demonstrated that children less than 1 year of age with CF have significantly lower relative abundance of *Bacteroides* spp. and higher relative abundance of *Escherichia coli* in their stool compared with children without CF (5, 7, 8, 12, 13, 21). Additionally, *Bacteroides* relative abundance does not increase in early childhood for pwCF (20).

*Bacteroides* are primarily beneficial symbiotic microbes that promote normal immune development but can also be opportunistic pathogens (22, 23). We hypothesized that a lack of *Bacteroides* may alter immune signaling in pwCF, leading to higher systemic inflammation and higher rates of clinical exacerbation. We have previously utilized an *in vitro* trans-well coculture system to demonstrate that *Bacteroides* can decrease the levels of IL-8, a pro-inflammatory cytokine, in CRISPR-modified CFTR−/− Caco-2 human intestinal epithelial cells (5, 24). Additionally, live bacteria were not required for the reduction of IL-8 levels, as supernatants from *Bacteroides* cultures also reduced the levels of this cytokine.

In this study, we utilized a larger panel of *Bacteroides* isolates and investigated the metabolomic and anti-inflammatory properties of these isolates. We found that IL-8 is broadly downregulated by many *Bacteroides* isolates and that this downregulation is dependent on the production of propionate. We next used a mouse model of CF, *Cftr^F508del^*, that is homozygous for the most common CF mutation in pwCF, to further explore the impact of *Bacteroides* on the CF microbiome and inflammatory state. The addition of *Bacteroides* isolates to the gut of *Cftr^F508del^* mice demonstrated that *Bacteroides* can persist and the presence of intestinal *Bacteroides* can decrease markers of systemic inflammation as well as shift the microbial populations of the fecal microbiota. Furthermore, a *Bacteroides* mutant deficient in propionate production failed to reduce pro-inflammatory cytokines despite reaching equivalent absolute abundance levels in the gut compared with its isogenic parent. This work demonstrates that *Bacteroides* may be a key driver of the gut-lung axis in CF and that the ability to produce propionate plays an important role in shaping the host inflammatory state as well as intestinal microbial ecology. Our work supports a role for *Bacteroides* as a potential probiotic for pwCF.

## RESULTS

### Supernatants from *Bacteroides* isolates downregulate IL-8

Previous work from our group utilized a CFTR−/− Caco-2 intestinal epithelial cell line developed by Hao et al. (24) to demonstrate that *Bacteroides* supernatants can downregulate the TNF-α-stimulated production of IL-8 in a trans-well coculture system (5). Previous studies demonstrated that Caco-2 intestinal epithelial cells cultured on plastic are more responsive to IL-1β stimulation than TNF-α or lipopolysaccharide (LPS) (25). We therefore tested the response of three CFTR−/− and three WT Caco-2 intestinal epithelial cell lines cultured on plastic to stimulation with 10 ng/mL IL-1β for 24 hours. IL-1β stimulated robust production of IL-8 in all cell lines (Fig. S1A and B) but with considerable variability dependent on both cell line and experimental replicate. All subsequent experiments were performed in the CFTR−/− S1 cell line as this line was used in our previous work (5) and showed a relatively consistent response in terms of IL-8 production when treated with IL-1β. A 24-hour time course of IL-1β stimulation of CFTR−/− S1 cells demonstrated that IL-8 accumulates rapidly over the first 6 hours of IL-1β exposure and then more slowly up to 24 hours (Fig. S1C).

Next, we tested the impact of supernatants derived from *Bacteroides* and *Parabacteroides* isolates on the IL-1β-stimulated IL-8 production by CFTR−/− S1 Caco-2 intestinal epithelial cells (hereafter called “CFTR−/− Caco-2 cells”). We isolated these *Bacteroides* and *Parabacteroides* strains from the stool of children with and without CF (Table S1). Bacterial supernatants were prepared from 19 isolates in supplemented minimal essential medium (sMEM; see Materials and Methods) and screened *in vitro* for their effects on the inflammatory response of IL-1β-stimulated CFTR−/− Caco-2 cells (Fig. S2A). The majority of the *Bacteroides* isolates trended toward downregulation of IL-8, while supernatants from *Parabacteroides* tended to have no effect or to increase IL-8 production. Supernatant treatment did not significantly lower the viability of the CFTR−/− Caco-2 cells, but treatment with *Parabacteroides* supernatant trended toward higher viability relative to IL-1β alone (Fig. S2B). Growth of *Bacteroides* and *Parabacteroides* varied in sMEM in an isolate-dependent manner (Fig. S2C). In general, *Bacteroides* grew to CFU 100-fold higher than *Parabacteroides* in sMEM, which may partially explain the stronger downregulatory effects on IL-8 observed for *Bacteroides*.

*Bacteroides* from persons with CF (pwCF) did not have significantly different impacts on IL-8 compared with *Bacteroides* isolates from healthy children (Fig. S3A). We found a modest negative correlation between *Bacteroides* growth and IL-8 production by simple linear regression (*R*^2^ = 0.075, *P* = 0.039), as well as a positive correlation between IL-8 production and CFTR−/− Caco-2 cellular viability (*R*^2^ = 0.103, *P* = 0.018; Fig. S3B and C). In summary, IL-8 downregulation is primarily dependent on isolate, is modestly influenced by CFTR−/− Caco-2 cellular viability and *Bacteroides* growth, and is independent of isolate origin.

**Fig 1:**
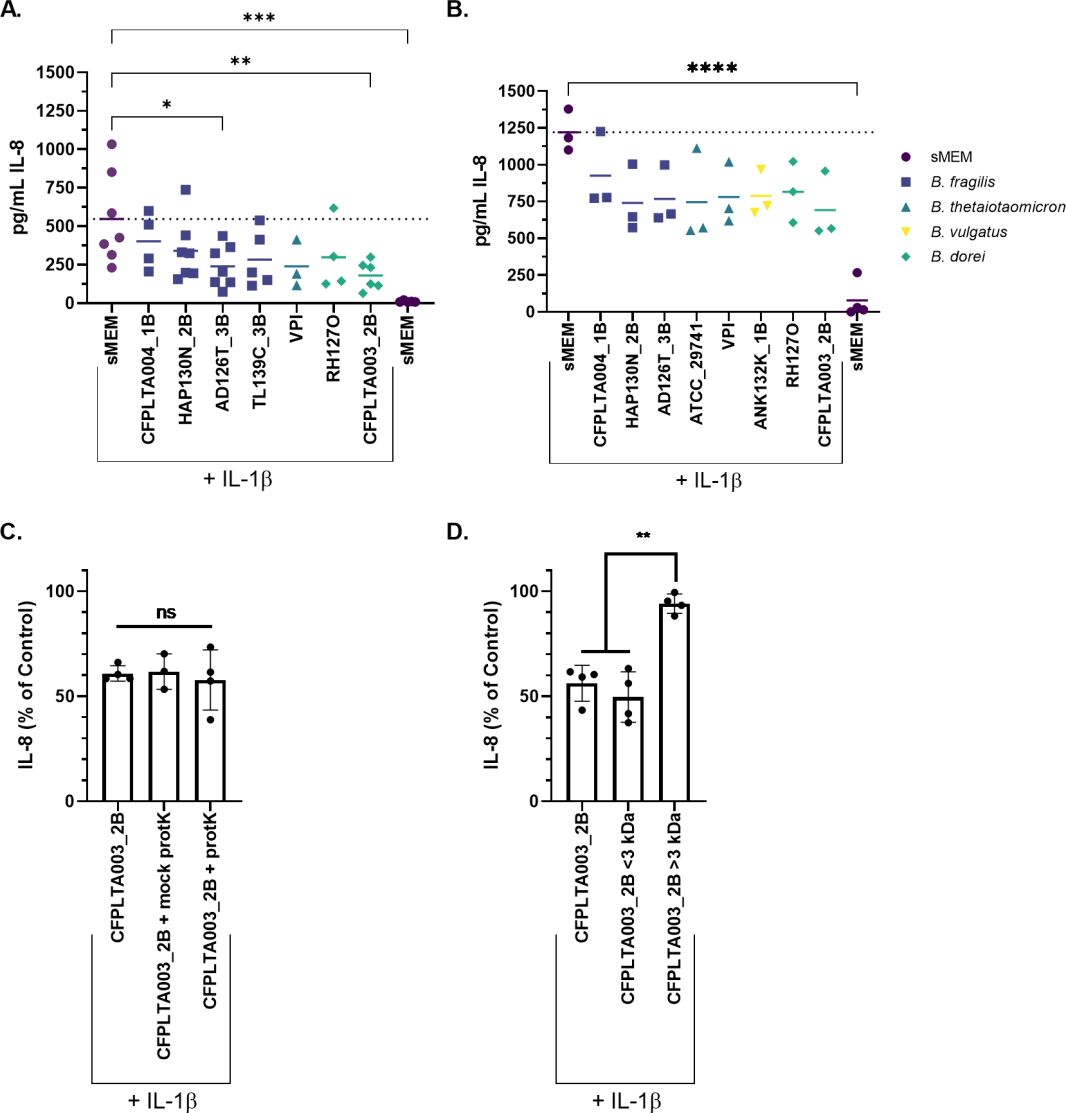
*Bacteroides* supernatants downregulate IL-8 production by Caco-2 cells, and IL-8 downregulation is likely not due to a protein. CFTR−/− Caco-2 cells were cultured for 2 weeks in 24-well plates and then exposed to *Bacteroides* supernatants prepared in sMEM as described in the Materials and Methods. Each point represents the average of 4–6 technical replicates from a single biological replicate. IL-8 was quantified by ELISA after (A) 6 hours and (B) 24 hours of CFTR−/− Caco-2 cell exposure to the bacterial supernatants from the indicated *Bacteroides* strains. Individual data points are shown, and the median is represented by the horizontal lines, and the dotted line is the median of the sMEM + IL-1β control. Significance was determined by unpaired one-way ANOVA followed by Dunnett’s post-test with sMEM + IL-1β as the reference. *P < 0.05, **P < 0.01, and ****P < 0.0001. (**C, D**) IL-8 was quantified by ELISA after 24 hours of CFTR−/− Caco-2 cell exposure to the bacterial supernatants from the indicated treatment. IL-8 quantity is displayed as a percentage of the sMEM + IL-1β control in the same biological replicate. Effects of (C) proteinase K treatment and (D) size fractionation on IL-8-reducing activity. Significance was tested by unpaired one-way ANOVA followed by Tukey’s post-test. **P < 0.01. Individual data points and the mean are shown.

A subset of *Bacteroides* isolates was selected, and supernatants from these strains were prepared and re-tested for their impact on IL-8 production by IL-1β-stimulated CFTR−/− Caco-2 cells grown in 24-well plates. A control with no IL-1β was included, showing that baseline IL-8 production is low (~10 ng/μL; Fig. 1A). IL-8 secreted from the CFTR−/− Caco-2 cells was measured by enzyme-linked immunosorbent assay (ELISA) after exposure to bacterial supernatants for 6 or 24 hours (Fig. 1A and B). We identified two isolates, *Bacteroides (Phocaeicola*) *dorei* CFPLTA003_2B and *Bacteroides fragilis* AD126T_3B, that had consistent, strong IL-8 downregulatory effects after 6 hours of coculture (Fig. 1A). The downregulatory effects of *Bacteroides* supernatants were reduced after 24 hours, but IL-8 still trended downwards in the presence of supernatants (Fig. 1B). Overall, *Bacteroides* supernatants reduce the level of IL-8 measured for CFTR−/− Caco-2 cells stimulated with IL-1β.

### The majority of *Bacteroides* isolates tested do not encode *Bacteroides fragilis* toxin

Enterotoxigenic strains of *Bacteroides fragilis* produce a protein known as *Bacteroides fragilis* enterotoxin (BFT) that is implicated in many intestinal inflammatory disorders, including the development of colorectal cancer (26, 27). BFT is located on a mobilizable pathogenicity island and thus has the potential to be transferred between isolates (28, 29). Furthermore, BFT can induce IL-8 production (30), which may interfere with IL-8-downregulating factors in our assay.

To rule out the possibility that BFT was interfering with IL-8 downregulation, we probed the genomes of our *B. fragilis* isolates for their potential to encode this toxin. Whole genome sequencing data are available for all but one of the isolates in this study (PRJNA557692). We used tblastn to assess each isolate for BFT (see Materials and Methods for details). Significant sequence similarity to BFT was identified in the isolate TL139C_3B. Interestingly, this isolate moderately downregulates IL-8 (Fig. S2A and B), indicating that if BFT is expressed under our experimental conditions, it does not eliminate the reduction in IL-8 mediated by this strain.

### The effects of *Bacteroides* supernatants on the IL-8 level is due to a heat-stable, small-molecular weight factor

We selected a single *B. dorei* isolate, CFPLTA003_2B, to work with for initial studies; this isolate was chosen for its consistent and strong reduction of the IL-8 level. We treated supernatants from *B. dorei* isolate CFPLTA003_2B with proteinase K (protK) to determine whether *Bacteroides-*mediated reduction of IL-8 was protein dependent (see Materials and Methods for details). Supernatant was treated with protK for 1–2 hours at 37°C, and protK was subsequently inactivated by heating at 95°C for 15 minutes in a sealed plastic tube. A mock condition, where supernatant underwent the same temperature incubation steps as the protK-treated samples, was also included. Supernatant from *B. dorei* CFPLTA003_2B downregulated IL-8 from standard, mock, and protK-treated conditions (Fig. 1C). The lack of sensitivity to protK and extended heat treatment indicated that a protein is likely not required for supernatant-mediated IL-8 reduction and that the factor(s) required for this activity are heat stable.

**Fig 2:**
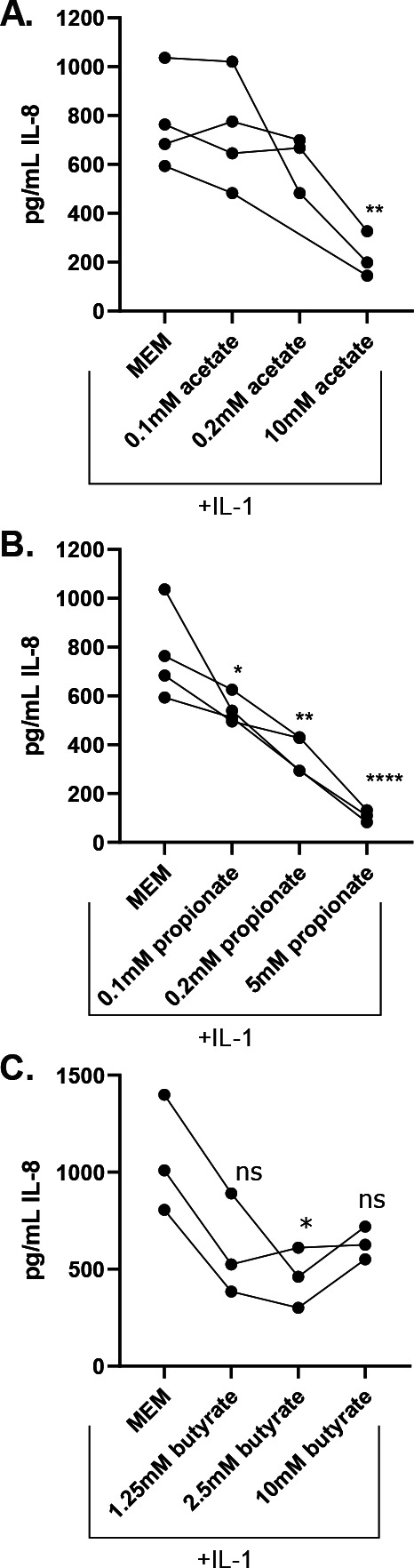
CFTR−/− Caco-2 cells are responsive to the anti-inflammatory effects of SCFAs. CFTR−/− Caco-2 cells were cultured for 2 weeks in 24-well plates and then treated with IL-1β alone or with the addition of the indicated concentrations of SCFAs. IL-8 was quantified by ELISA after 24 hours of culture with (A) sodium acetate, (B) sodium propionate, or (C) sodium butyrate at the indicated concentration. Each point indicates the mean of 4–6 technical replicates from a single biological replicate, with 3–4 independent biological replicates performed. Lines connect results from experiments performed on the same day. Significance was tested by unpaired one-way ANOVA followed by Dunnett’s post-test with MEM + IL-1β as the reference. *P < 0.05, **P < 0.01, and ****P < 0.0001.

We next used a 3-kDa-size cut-off filter to estimate the size of the factor(s) responsible for IL-8 reduction. *B. dorei* CFPLTA003_2B supernatant was placed in a 3-kDa spin column, and CFTR−/− Caco-2 cells were incubated with either the flowthrough containing small-molecular weight molecules (<3 kDa) or the retentate fraction containing large molecules > 3 kDa, which was subsequently diluted to its original volume. The flowthrough, but not the retentate fraction, reduced the IL-8 level (Fig. 1D), supporting the conclusion that a small-molecular weight molecule was responsible for this activity.

### IL-8 production by the CFTR−/− Caco-2 cell line is responsive to SCFAs

Short-chain fatty acids (SCFAs) are generated by microbial fermentation in the intestine and are an important aspect of the microbiome that is beneficial to host health, as SCFAs both provide an important source of energy for colonic epithelial cells and regulate inflammatory host responses (31). The three highest-abundance SCFAs in the intestine are acetate, propionate, and butyrate (31). *Bacteroides* have been reported to produce the two SCFAs propionate and acetate but not butyrate (32). We hypothesized that *Bacteroides*-derived propionate or acetate might be reducing IL-8 in the coculture experiments (see Fig. 1). We tested the dose responsiveness of these three abundant SCFAs and found that CFTR−/− Caco-2 cells respond to all three, albeit at different concentrations (Fig. 2).

The CFTR−/− Caco-2 cells are responsive to acetate starting at 10 mM (Fig. 2A) but are very responsive to propionate at a concentration as low as 0.1 mM (Fig. 2B). Neither acetate nor propionate impact cellular viability at the concentrations tested (Fig. S4A and B). The IL-8 inflammatory response is not linearly responsive to butyrate; downregulation occurs at 2.5 mM but not at 10 mM; this observation is likely because butyrate begins to inhibit cellular viability at a 10-mM concentration (Fig. S4C). In summary, CFTR−/− Caco-2 cells are responsive to the anti-inflammatory effects of major intestinal SCFAs.

### The supernatant from a strain of *Bacteroides thetaiotaomicron* deficient in propionate production exhibits diminished anti-inflammatory activity compared with a wild-type strain

We utilized *B. thetaiotaomicron* BT1686-89, a mutant lacking the methyl malonyl-CoA transcarboxylase gene, which is required for the conversion of succinate to propionate and is deficient in propionate production, to test whether propionate is necessary for the downregulation of IL-8 observed for *Bacteroides* supernatants (33). BT1681-89 was constructed in the *B. thetaiotaomicron* VPI-5482 Δ*tdk* background and is referred to as *B. thetaiotaomicron* Δ*prp* or simply Δ*prp* in this report. The wild-type *B. thetaiotaomicron* VPI-5482 and *B. thetaiotaomicron* Δ*tdk* isogenic parents are both included as controls here. Supernatants were prepared from each strain in sMEM, and the supernatants were applied to the CFTR−/− Caco-2 cells for 6 and 24 hours as described in the Materials and Methods.

**Fig 3:**
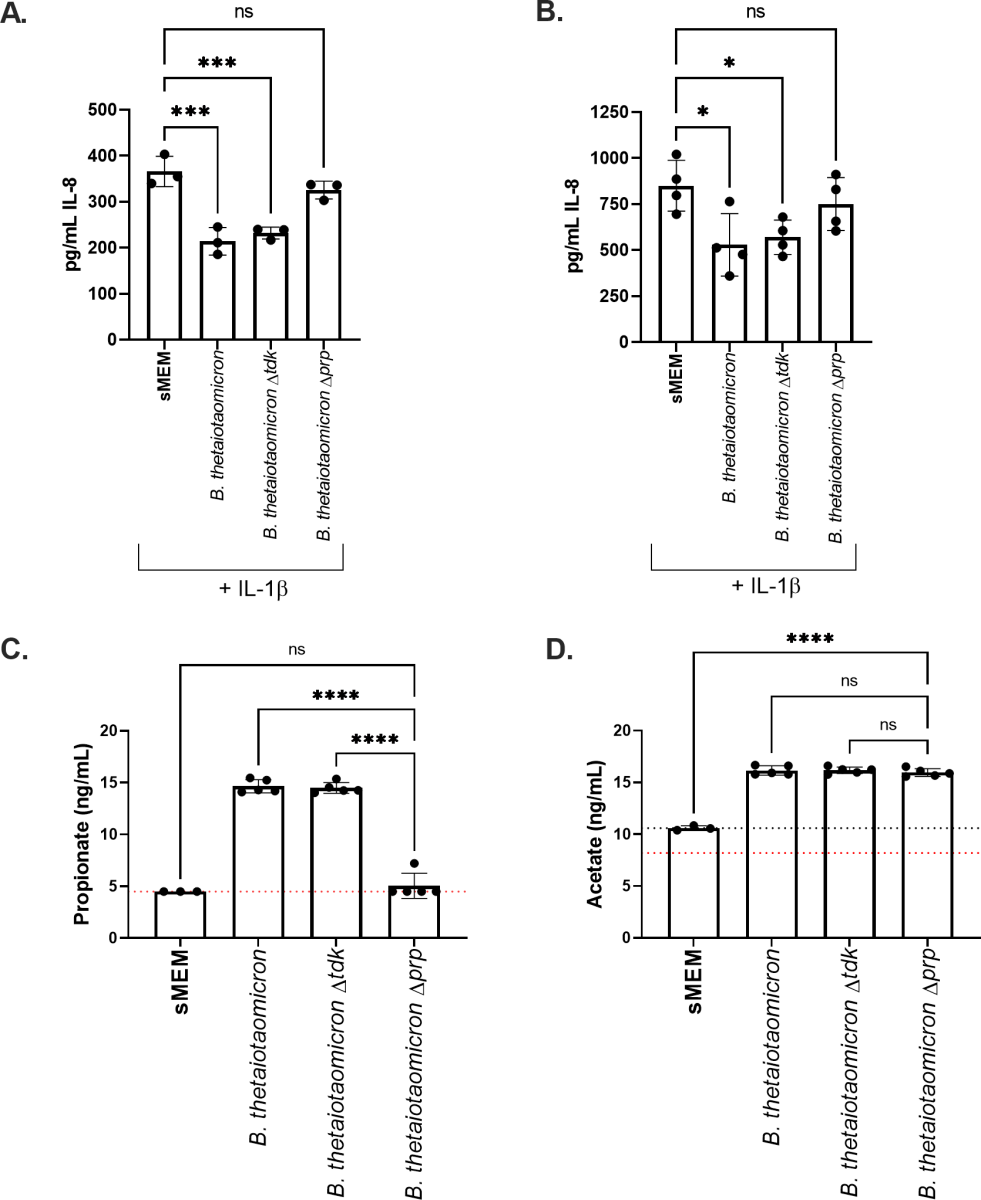
IL-8 downregulation by *B. thetaiotaomicron* is propionate dependent. CFTR−/− Caco-2 cells were cultured for 2 weeks in 24-well plates and then incubated with *B. thetaiotaomicron* supernatants from the indicated strains prepared in sMEM as described in the Materials and Methods. IL-8 was quantified by ELISA after (A) 6 hours and (B) 24 hours of the CFTR−/− Caco-2 cell exposure to the bacterial supernatants from the indicated strains. Each point represents the average of 4–6 technical replicates from a single biological replicate, with three independent biological replicates performed. Significance was determined by unpaired one-way ANOVA followed by Dunnett’s post-test with sMEM + IL-1β as the reference. *P < 0.05, ***P < 0.001 and ****P < 0.0001. The supernatants in these assays were not normalized to cell density, but there is no difference in the growth of the strains in this medium (see Fig. S5A). The SCFAs propionate (panel C) and acetate (panel D) were quantified by gas chromatography - mass spectrometry (GC-MS) (ng/μL) and log2-transformed. The dashed red line indicates the assay limit of detection (LOD). In panel D, the dashed black line indicates the average acetate concentration detected in the medium, as this medium contains acetate above the LOD. Significance was determined by unpaired one-way ANOVA followed by Dunnett’s post-test with *B. thetaiotaomicron* Δ*prp* as the reference. ns = not significant, ****P < 0.0001. In all panels, individual data points are shown and the median is represented by the horizontal line.

While supernatants from both wild-type *B. thetaiotaomicron* and *B. thetaiotaomicron* Δ*tdk* strains significantly reduced the IL-8 level at 6 and 24 hours, the *B. thetaiotaomicron*Δ*prp* did not show such activity (Fig. 3A and B). Each bacterial culture was also plated for CFU/mL, and there was no significant difference between any of the three strains when cultured in sMEM (Fig. S5A), indicating that differential growth of the strains was not responsible for the observed impact on the IL-8 level. XTT assays were performed for each experiment to assess the viability of the CFTR−/− Caco-2 cells after treatment with the supernatants for 6 or 24 hours; there was no difference in viability among the strains caused by treatment with the supernatant (Fig. S5B and C).

SCFAs were also quantified from supernatants from each strain by GC-MS, confirming that *B. thetaiotaomicron* Δ*prp* did not produce propionate but the WT and Δ*tdk* strains did produce this SCFA and that the quantity of other measured SCFAs was not altered by the loss of the methyl malonyl-CoA transcarboxylase gene and the resulting loss of propionate production (Fig. 3C and D; Fig. S5D through G). Together, these results demonstrate that *Bacteroides*-mediated downregulation of IL-8 is dependent on propionate production.

### Propionate production is highly conserved across *Bacteroides* isolates

**Fig 4:**
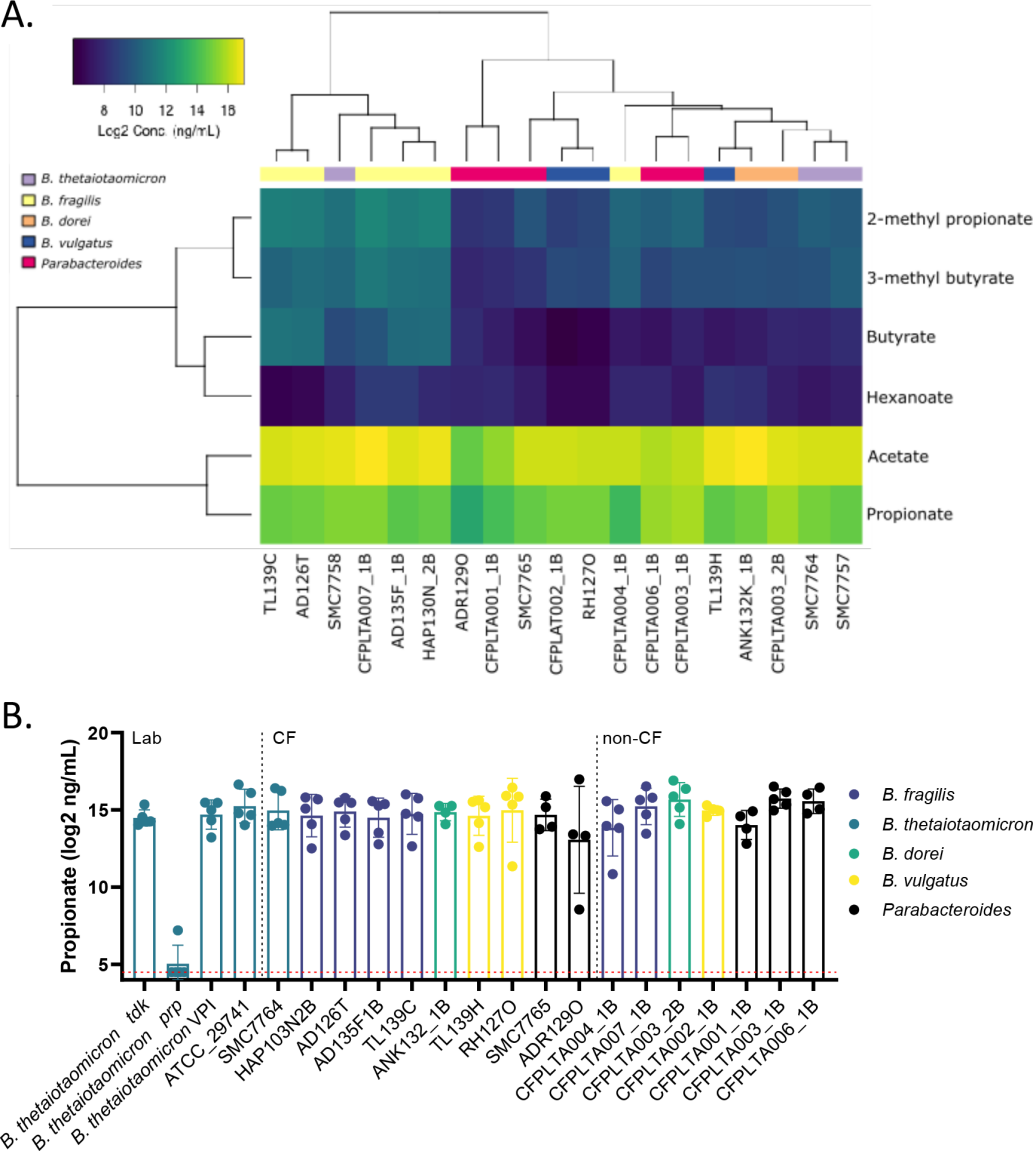
Strain-dependent production of SCFA and IL-8 downregulation. SCFAs were quantified in undiluted, sterile-filtered supernatants by GC-MS as described in the Materials and Methods. (**A**) Heatmap of log2-transformed concentration (ng/mL) of each detectable SCFA. Pentanoic and 2-methyl pentanoates were not detected above the LOD. (**B**) Propionate produced by individual isolates. The dashed red line indicates the LOD.

We next determined whether there were strain-dependent differences in SCFA production by *Bacteroides* isolates. We therefore prepared supernatants from the 19 isolates originally screened for IL-8 downregulation (Fig. S2) and quantified SCFAs in each supernatant by GC-MS. Unsurprisingly, acetate and propionate were the primary SCFAs produced by all isolates of *Bacteroides* and *Parabacteroides* (Fig. 4A; Fig. S6A and B). Other detectable SCFAs at concentrations above the medium control included 2-methyl propionate, 3-methyl butyrate, and hexanoate (Fig. S6C through F). Pentanoate and 2-methyl pentanoate were not present above the limit of detection, and heptanoate and butyrate were not routinely detected above the medium control.

Clustering of SCFAs demonstrated partial separation by species, with a distinct *B. fragilis* signature (Fig. 4A). We detected species-specific differences in acetate, 2-methyl propionate, and 3-methyl butyrate, all of which were highest in *B. fragilis* isolates and lowest among *Parabacteroides* isolates (Fig. S6). At the isolate level, the *B. dorei* CFPLTA003_2B isolate produced the highest concentration of propionate (Fig. 4B; Fig. S6B). However, this concentration was not statistically significantly higher than other supernatants, suggesting that propionate is necessary but not sufficient to explain the robust anti-inflammatory properties of CFPLTA003_2B.

### *Bacteroides* species-level metabolite signatures

The identification of multiple SCFAs of interest raised the question of whether other metabolites produced by *Bacteroides* might impact the regulation of the inflammatory response by intestinal epithelial cells. We used LC-MS to generate metabolomic profiles of several *Bacteroides* isolates cultured in MEM for 48 hours or cultured in sMEM for 24 hours. Clustering of metabolite profiles demonstrated that *Bacteroides* produced significantly more metabolites in MEM versus sMEM, likely due to increased culture time (48 hours vs. 24 hours, respectively) under these conditions (Fig. S7A). Metabolites were measured for four isolates in MEM, and individual biological replicates clustered well at the isolate and species level (Fig. S7B). PCA plots of all biological replicates demonstrated clustering by isolate, with the *B. dorei* CFPLTA003_2B and *B. vulgatus* RH127O isolates clustering more closely than the two *B. thetaiotaomicron* isolates (Fig. S7C).

LC-MS analysis of the metabolites produced by isolates cultured in sMEM demonstrated higher variability, with individual biological replicates not clustering well together (Fig. S8A). However, a PCA plot of all metabolites detected demonstrated that there was some species-level separation, with *B. thetaiotaomicron* separating from *B. fragilis* and *B. vulgatus*/*B. dorei* (Fig. S8B).

We identified 24 metabolites with at least a threefold increase in every supernatant tested over the medium control (Fig. S9A). The three metabolites with the highest average relative abundance increase were lactate, malate, and succinate. We tested each of these metabolites alone and found that lactate modestly downregulated the IL-8 level at concentrations of 5 mM or greater, while neither succinate nor malate downregulated IL-8. Indeed, malate enhanced the IL-8 level at high concentrations (Fig. S9B).

We next screened a panel of a total of 26 commercially available metabolites identified by liquid chromatography - mass spectrometry (LC-MS) in the *Bacteroides* supernatants (Fig. S9C). Most of the metabolites had no effect or increased IL-8 production, or if they did reduce IL-8 production, this impact was due to their cytotoxic effects versus the CFTR−/− Caco-2 cells (Fig. S9C). Only one compound of the 26 tested, riboflavin, showed a measurable reduction of IL-8 production with no detectable cytotoxicity versus the CFTR−/− Caco-2 cells (Fig. S9C). Thus, future studies are warranted to address the impact of riboflavin to modulate IL-8 production at physiological concentrations. Taken together with the analysis above, propionate appears to be the major metabolite driving the reduction in IL-8 production.

### Supplementing *Bacteroides* isolates alters SCFAs and cytokine production in a *Cftr^F508del^* mouse model

To test the impact of the addition of *Bacteroides* to the CF gut microbiome, we designed a mouse experiment wherein the native gut microbiome of specific pathogen-free *Cftr^F508del^* mice [also called *Cftr^em1Cwr^* (34), which carry the most common CFTR allele] was first suppressed by sustained antibiotic treatment, and then, the microbiota was replaced by oral gavage with a stool pool from children with CF. The stool pool contained either the three *Bacteroides* isolates *B. dorei* CFPLTA003_2B, *B. thetaiotaomicron* ATCC 29741, and *B. fragilis* AD126T 3B (see Fig. 1; Fig. S2A; Tables S1 and S2) or phosphate buffered saline (PBS) only as a control, such that the two conditions were mice with a CF-like gut microbiota with and without the presence of added *Bacteroides* strains. Mouse stool was collected prior to the beginning of antibiotic treatment and for 2 weeks post-gavage for analysis of 16S rRNA gene-based microbiome structure, cytokines, IgA, and SCFA content (Fig. S10A). Two weeks after introducing the stool pools, mice were intranasally challenged with 0.2 mg/kg *Pseudomonas aeruginosa* LPS and then sacrificed 24 hours post-challenge. Before sacrificing, fecal pellets were collected. At the time of sacrificing, serum was collected for analysis of cytokines and SCFAs, and intestines were collected for analysis of cytokines, SCFAs, and microbiology. Additionally, lung and intestinal tissues were collected for analysis of the inflammatory response via hematoxylin and eosin stain (H&E) staining. Evaluation of gastrointestinal inflammation was completed by two pathologists with blinded data sets based on previously established scoring rubrics for evaluation of intestinal inflammation in mouse models (35). Lung inflammation was evaluated by two pathologists with blinded data sets and scored using a previously established scoring system for lung injury in CF mice (36). Scores were averaged, and statistical significance was determined using a linear model on log2-transformed average scores of the lungs (non-normally distributed data) and non-transformed average scores of the normally distributed intestinal data, accounting for treatment, experiment, cage, and sex of mouse.

**Fig 5:**
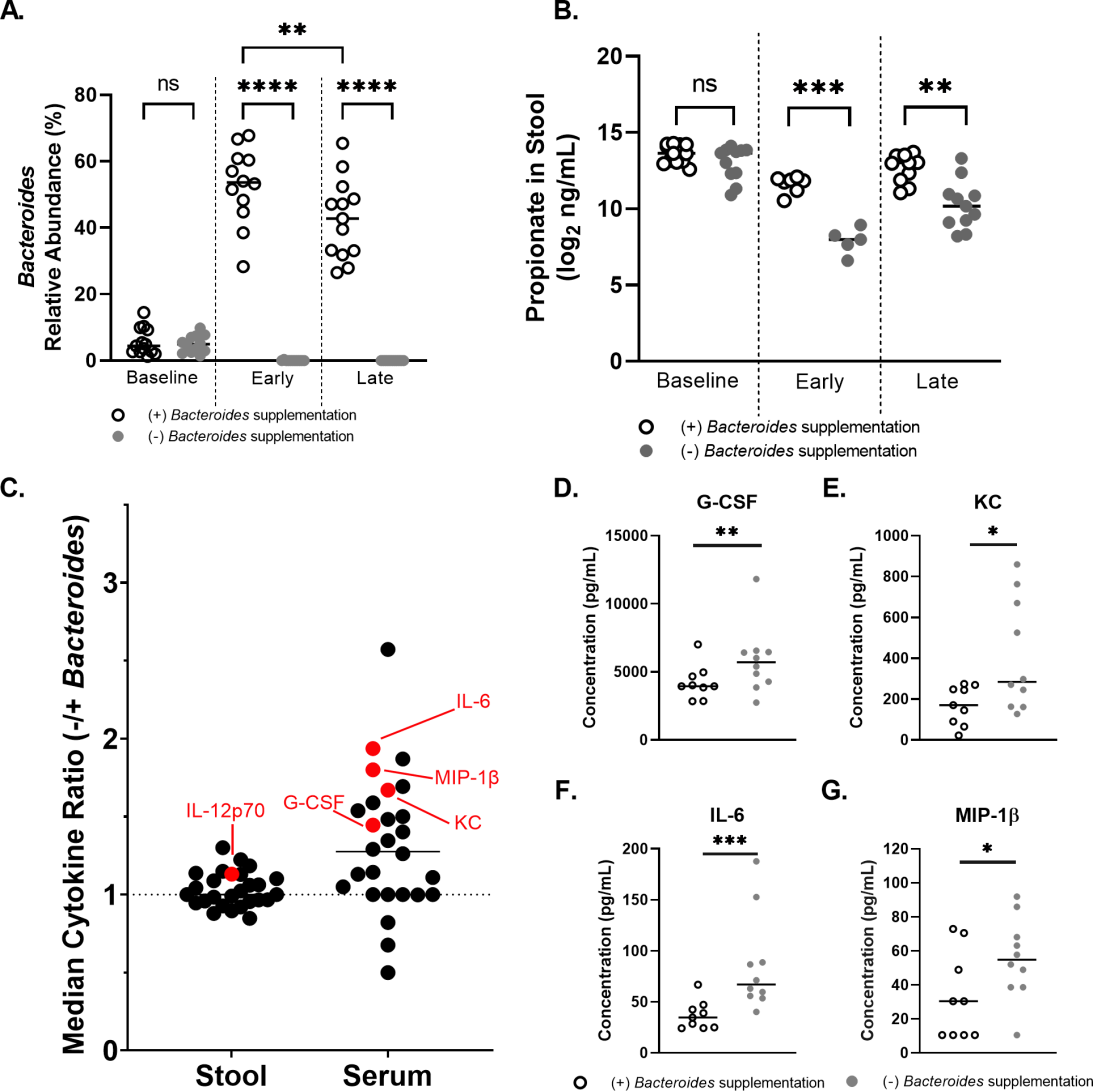
A *CftrF508del* mouse model reveals that intestinal supplementation of *Bacteroides* reduces systemic cytokine levels. (**A-C**) Stool samples were collected from *CftrF508del* mice either before the onset of antibiotic treatment (baseline) or post-gavage with pooled CF stool ± supplemented *Bacteroides*. Each point represents data from a single mouse stool in all graphs. (**A**) Microbial composition of stool was profiled for each mouse by 16S rRNA gene amplicon sequencing to determine *Bacteroides* relative abundance. “Early” indicates that the stool was collected 2–4 days post-gavage and “Late” indicates collection 12–13 days post-gavage. (**B**) Propionate was quantified by GC-MS from mouse stool samples during the early or late windows (days 1 & 9, respectively). (**A, B**) Statistical significance was analyzed by linear model on log2-transformed propionate concentrations, accounting for ±supplemented *Bacteroides*, experiment, cage, and sex of mouse *P < 0.05, **P < 0.01, and ***P < 0.001. (**C**) Fecal pellets, then serum, and intestine were collected at the end of the experiment 24 hours after LPS challenge. A complete timeline of the experiment is shown in Figure S10A. Cytokines are displayed as a ratio of the median cytokine concentration in the (−) supplemented *Bacteroides* condition divided by the median cytokine concentration in the (+) supplemented *Bacteroides* condition. Cytokines were quantified by Luminex 32-plex panel for the indicated cytokines. Statistical significance was analyzed by linear model on log2-transformed cytokine concentrations, accounting for ±supplemented *Bacteroides*, experiment, cage, and sex of mouse with *P* < 0.05 labeled in red, and indicate higher levels for the (−) supplemented *Bacteroides* condition. (**D-G**) Serum cytokines quantified by Luminex that were statistically significant between the (+) and (−) supplemented *Bacteroides* conditions by linear model on log2-transformed cytokine concentrations, accounting for ±supplemented *Bacteroides*, experiment, cage, and sex of mouse. *P < 0.05 and **P < 0.01. When the high concentration outlier > 10,000 pg/mL in the (−) supplemented *Bacteroides* G-CSF measurement is removed, this result remains significant with *P* = 0.0243. After we account for multiple comparisons, by Bonferroni correction, only IL-6 is significant. (**D**) G-CSF (granulocyte colony stimulating factor), (**E**) KC (CXCL1; IL-8 homolog), (F) IL-6 (Interleukin 6), and (**G**) MIP-1β (macrophage inflammatory protein 1β). Horizontal bars in each graph display the median.

Analysis of the 16S rRNA gene amplicon data across all samples showed that the average *Bacteroides* relative abundance was 5.4% at baseline (that is, before any treatments; Fig. 5A; Fig. S10B). CF stool pools used to gavage mice for the (+) and (−) supplemented *Bacteroides* groups contained a mean relative abundance of 83.4% and 0.33% *Bacteroides*, respectively (Fig. S10B, stool pool). Post-gavage *Bacteroides* relative abundance was 47.4% for the (+) *Bacteroides* condition and 0.02% for the (−) *Bacteroides* condition (Fig. S10B, samples).

Fecal samples collected for amplicon sequencing within 2–4 days post-gavage were grouped as “early,” and those collected on day 12 or 13 post-gavage were grouped as “late.” *Bacteroides* relative abundance did not significantly change between early and late samples in the (−) *Bacteroides* condition (Fig. 5A). *Bacteroides* relative abundance remained significantly higher than baseline throughout the post-gavage period but decreased from an average relative abundance of 53.6% early to 42.8% late in the (+) *Bacteroides* condition (Fig. 5A).

The overall community structure of these samples was analyzed by multidimensional scaling ordination based on Bray-Curtis dissimilarity coefficients (Fig. S10C). The composition of the (+) and (−) *Bacteroides* conditions was significantly different from each other as determined by permutational multivariate analysis of variance (PERMANOVA) (*P* = 0.014) early post-gavage and not significantly different (*P* = 0.085) during the late window post-gavage. The community was further analyzed at both time points at the genus level for significantly altered relative abundance of amplicon sequence variants (ASVs) by DESeq2 (Fig. S10D and E). Interestingly, few ASVs were significantly different between the two conditions and only three were altered in the same direction at both time points. Unsurprisingly, the relative abundance of *Bacteroides* was higher in the (+) *Bacteroides* condition in both the early and late windows. Both *Parabacteroides* (Fig. S10D and E) and *Escherichia* (Fig. S10E and F) were also significantly reduced in the (+) supplemented *Bacteroides* relative to the (−) supplemented *Bacteroides* condition in both the early and late windows. These data show that the *Bacteroides* spp. introduced by gavage were effectively retained during the course of the ~2-week experiment and may influence the abundance of key CF-associated intestinal microbes, specifically *E. coli*. However, introduction of *Bacteroides* did not radically alter the overall stool microbiota composition, particularly at the later time points. These data align with previous clinical studies showing specific changes in the microbiota of infants and children with CF (5, 7, 11, 13, 18–20).

The presence of *Bacteroides* significantly increased the amount of detectable propionate, 2-methyl propionate, and 3-methyl butyrate but not acetate or butyrate in the stool (Fig. 5B; Fig. S11).

Next, to assess whether the presence of *Bacteroides* and subsequent changes in local SCFA concentrations impacted either local or systemic inflammation, we quantified both stool and serum cytokines post LPS exposure using a Luminex 32-plex cytokine panel (Fig. 5C through G; Fig. S12 to S13). Significant changes between conditions were tested on log2-transformed concentrations by linear model, and the average ratio of cytokines in the (−) supplemented *Bacteroides* condition divided by the (+) supplemented *Bacteroides* condition was graphed for each cytokine (Fig. 5C). Interestingly, 19 of the 29 cytokines in stool above the level of detection trended higher in the (−) supplemented *Bacteroides* condition. Only one cytokine, IL-12 (p70), was significantly different between conditions, and it was higher in the (−) supplemented *Bacteroides* condition (Fig. S12).

The majority of serum cytokines followed a similar pattern, with 23 of the 29 cytokines detected trending higher in the (−) supplemented *Bacteroides* condition (Fig. S13). Four pro-inflammatory cytokines were significantly altered in serum, all of which [G-CSF, KC (CXCL1; IL-8 homolog), IL-6, and MIP-1b] were higher in the (−) supplemented *Bacteroides* condition (Fig. 5D through G).

Local and systemic inflammation was also assessed by quantification of calprotectin and IgA in the stool (Fig. S14) and analysis of H&E-stained lung and intestinal tissue (Fig. S15), but no significant differences were detected between conditions.

To assess whether higher local SCFAs corresponded with higher systemic SCFAs, we also quantified 12 SCFAs in the serum collected at the time of sacrificing. Only 3-methyl valerate, which was modestly higher in the (+) supplemented *Bacteroides* condition, was significantly altered in the serum (Fig. S16).

Overall, these data demonstrate that *Bacteroides* in the gut lead to higher intestinal but not systemic propionate, shifted the microbial community in the gut (specifically, reduced relative abundance of *E. coli*), and had a greater impact on systemic (serum) pro-inflammatory cytokine levels than local (fecal) inflammatory marker levels, a result that has implications for the role of *Bacteroides* in the gut-lung axis in CF.

### *Cftr^F508del^* mouse model reveals that propionate is required for *Bacteroides* reduction of airway cytokine levels

To investigate the role of *Bacteroides*-synthesized propionate in host inflammatory response, we tested whether the propionate-deficient *B. thetaiotaomicron* Δ*prp* mutant would influence systemic inflammation or protect *Cftr^F508del^* mice from LPS-induced airway inflammatory response. Using a similar experimental design as described above and in the Materials and Methods, mice were gavaged with a CF stool pool supplemented with either *B. thetaiotaomicron* Δ*tdk* or *B. thetaiotaomicron* Δ*prp*. Two weeks after gavage, we challenged the mice with nasally administered 0.2 mg/kg *P*. *aeruginosa* LPS. Twenty-four hours after LPS challenge, mice were sacrificed and the following were collected and analyzed for their cytokine profiles by a Luminex 32-cytokine panel: fecal pellets, then serum, lung tissue, and intestinal tissue.

**Fig 6:**
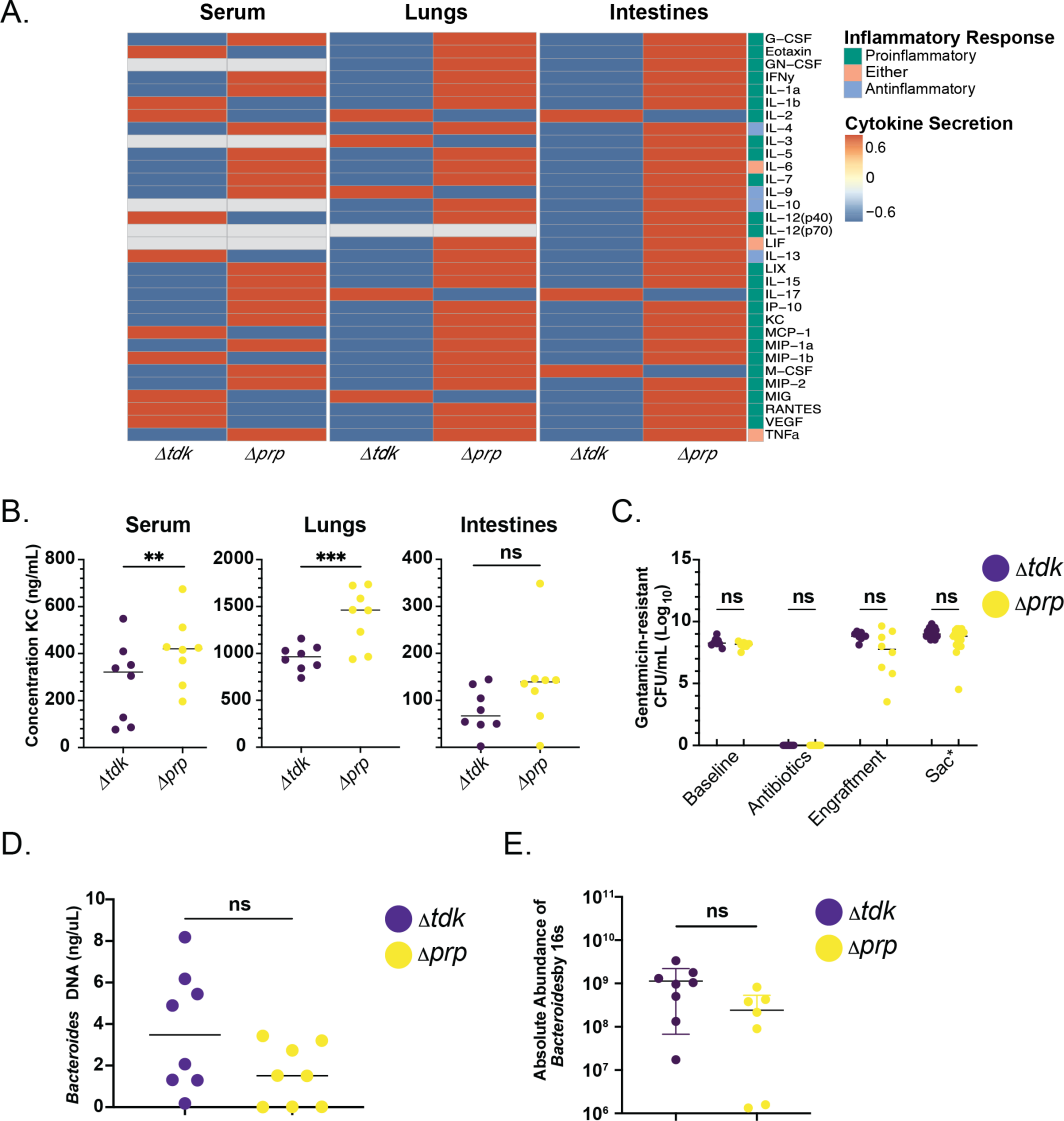
*Cftr^F508del^* mouse model reveals that intestinal supplementation of *Bacteroides* propionate mutant no longer reduces systemic cytokine levels. (**A**) Heatmap of normalized cytokine levels quantified by Luminex 32-panel in serum (left), lungs (middle), and colon (right). Mice were gavaged with CF stool + supplemented *Bacteroides*Δ*tdk* or CF stool + supplemented *Bacteroides*Δ*prp*. Samples were collected at sacrifice 24 hours after LPS challenge. Cytokines associated with a pro-inflammatory response are labeled in green, anti-inflammatory response are labeled in light-blue, and cytokines associated with either response are labeled in pink. Higher z-score levels of respective cytokine are indicated in red, and those lower are indicated in blue. Gray indicates that the cytokine was below detection. (**B**) Serum (left), lung (middle), and intestinal (right) KC cytokine levels quantified by Luminex 32-panel. Statistical significance was determined by linear model on log2-transformed KC concentrations, accounting for strain and sex of mouse. *P < 0.05, **P < 0.01, and ***P < 0.001. (**C**) Stool was collected throughout the experiment from mice gavaged with *Bacteroides* Δ*tdk* (purple) and *Bacteroides* Δ*prp* (yellow). Stool was serially diluted and plated to count CFU/mL on blood agar + 100 μg/mL gentamicin. Each point indicates stool collected from a single mouse. Upon sacrifice (Sac), intestines were dissected to isolate 3 regions: the cecum, proximal colon, and distal colon. The luminal contents of these three regions were plated for CFUs and make up the CFUs under Sac**, which is a sum of the CFU from the three regions of the intestine assessed (see Fig. S17A). Statistical significance was determined by Kruskal-Wallis with Dunn’s multiple pair-wise comparisons test. (**D**) Absolute abundance was determined by qPCR using DNA extracted from stool with *B. thetaiotaomicron* VPI-5482-specific probes to quantify establishment of strains *Bacteroides* Δ*tdk* (purple) or *Bacteroides* Δ*prp* (yellow). Statistical significance was determined by linear model for *B. thetaiotaomicron* DNA (ng/μL), accounting for strain, cage, and sex of mouse. ns, not significant. (**E**) Absolute abundance was measured by 16S rRNA gene amplicon sequencing from DNA extracted from stool spiked with ZymoBIOMICSSpike-In Control I. Statistical significance was determined by linear model for *Bacteroides* absolute abundance accounting for strain, cage, and sex of mouse. ns, not significant.

Consistent with the observed gut-lung axis in clinical studies, our analysis revealed that mice gavaged with the *B. thetaiotaomicron* Δ*prp* mutant had higher systemic and pulmonary cytokine levels, including KC, as compared with mice gavaged with the *B. thetaiotaomicron* Δ*tdk* parental strain (Fig. 6A, left and center panels, and Fig 6B; Tables S3 and S4). Additionally, intestinal tissue showed a trend of increased cytokine levels in the mice gavaged with *B. thetaiotaomicron* Δ*prp* versus the parent strain (Fig. 6A, right panel, and Fig 6B). Short-chain fatty acid quantification of stool by GC-MS revealed no significant differences in propionate, acetate, or butyrate levels at any time point (data not shown).

To assess the establishment of the *Bacteroides* strains in the intestinal lumen and mucosa of the mice, respectively, we plated stool and intestinal samples on blood agar supplemented with 100 μg/mL gentamicin, a standard method for measuring *Bacteroides* abundance in mouse models (37). Consistent with a previous study (37), we found that there were significant differences neither in CFU based on the analysis of fecal samples (Fig. 6C) nor across different regions of the colon (Fig. S17A; Table S4) at sacrifice. We noted a reduction in final OD value for the *B. thetaiotaomicron* Δ*prp* mutant compared with the parental strain grown *in vitro* in BHIS medium (Fig. S17B), but we observed no marked difference in attachment of these strains to CFTR−/− Caco-2 colonic epithelial cells (Fig. S17C and D). Finally, we note no difference in the absolute abundance of the WT versus *prp* mutant using two different assays (Fig. 6D and E), despite a shift in the relative abundance of these strains at later time points in the experiment (Fig. S17E).

## DISCUSSION

We have shown here that *Bacteroides*-secreted products downregulate the inflammatory response of CFTR−/− Caco-2 intestinal epithelial cells and that the production of propionate is required for this downregulation. With the exception of succinate, deletion of the gene required for propionate production did not alter the production of any other major SCFAs. We cannot rule out that the increased succinate (a precursor of propionate) in turn may affect production of other metabolites (33). However, succinate itself had no impact on IL-8 production *in vitro*. The finding that CFTR−/− Caco-2 cells are responsive to the anti-inflammatory effects of the three major SCFAs indicates that increasing intestinal SCFA concentrations, through either remediation of the gut microbiome or direct supplementation with SCFAs, has the potential to ameliorate systemic inflammation for pwCF. The data presented here regarding the impacts of propionate are also consistent with previous clinical findings. For example, both *Bacteroides* and propionate are decreased in the stool of pwCF (5, 12, 13, 20, 38). Furthermore, pwCF are known to have higher levels of intestinal inflammation, and propionate has been previously linked to decreases in the inflammatory response in non-CF intestinal epithelial cells (12, 31, 39–41).

Interestingly, the *Bacteroides* isolates that we worked with here produced similar levels of propionate but showed differential capacities for IL-8 downregulation, indicating that propionate is not the sole contributor to IL-8 downregulation. We have identified two additional metabolites, lactate and riboflavin, which are detected in *Bacteroides* supernatants and have IL-8 downregulatory effects. Thus, it is possible that a combination of these metabolites (and others) contributes to the anti-inflammatory effects of *Bacteroides. Bacteroides* have been identified as major intestinal producers of propionate, whereas members of the phylum Firmicutes are responsible for the bulk of intestinal butyrate production (42). Interestingly, several butyrate-producing taxa, including *Faecalibacterium*, *Roseburia*, and *Bifidobacterium*, also have decreased relative abundance in the gut of pwCF (17).

We have hypothesized a role for *Bacteroides* in local intestinal SCFA production and/or modulating systemic inflammation and/or lung health outcomes in CF. We therefore designed a mouse study to directly test these hypotheses whereby the native mouse microbiome in *Cftr^F508del^* mice was depleted through antibiotic treatment and then replaced by oral gavage with a pooled stool sample from children with CF. We have shown here that it is possible to replace the native mouse microbiome with a CF-like microbiome over ~2 weeks and that when this microbiome is supplemented with *Bacteroides* isolates, these microbes will be maintained at high abundance for at least 2 weeks. This latter finding is important if we want to consider *Bacteroides* supplementation as a viable probiotic strategy. We chose to use an antibiotic suppression model over a germ-free model for a number of reasons, the most important of which is that we wanted to address the proof of concept for future probiotic studies, which would involve supplementing these microbes in pwCF who already have an established gut microbiota.

As predicted from our *in vitro* studies, the introduction of *Bacteroides* resulted in an increase in measurable stool propionate. The mice with high *Bacteroides* also had significantly lower *systemic* levels of the pro-inflammatory cytokines KC (CXCL1; IL-8 homolog), IL-5, IL-6, and MIP-1β. In this model, we did not see the effects on gross airway inflammation as measured by H&E staining. Notably, while supplementation of mouse gut microbiota with *Bacteroides* resulted in higher levels of propionate in the stool, the non-supplemented mice still produced detectable propionate despite low measured levels of *Bacteroides* by 16S rRNA gene sequencing. These data raise interesting implications that need to be more fully explored. First, while propionate drives reduction of IL-8 *in vitro*, as discussed above, we identified several other metabolites produced by *Bacteroides* that have a similar activity. Thus, it may be a suite of metabolites produced by *Bacteroides* spp. that together most effectively modulate systemic production of cytokines. It is also possible that *Bacteroides* spp. occupy a specific spatial niche that allows these microbes to effectively communicate with the immune machinery, that is, the propionate we detected in the (−) supplemented *Bacteroides* condition in our analysis is not produced at the right time and/or in the right place by the other members of the microbiota to influence serum cytokine levels. An alternative explanation is that the effects of propionate on IL-8 production observed *in vitro* and the impact on reduced KC levels *in vivo* could occur via two different mechanisms—a more detailed understanding of how intestinally produced propionate impacts systemic inflammation in mice could help shed light on these observations.

We noted above that while we can detect higher stool propionate in mice supplemented with *Bacteroides*, we do not observe an increase of this SCFA in the serum. This is not unexpected, as propionate enters the portal vein and is metabolized in the liver such that only low concentrations of propionate are typically detectable in the periphery (42). In contrast, in mice supplemented with *Bacteroides*, most of the observed impacts on cytokines were in the serum. An important implication of this finding is that it argues against a model whereby circulating SCFA are acting to impact the host. One simple model to reconcile these findings is that *Bacteroides* acts locally in the gut to modulate serum cytokine levels and thus systemic inflammation.

Interestingly, the absence of *Bacteroides* was associated with a significantly higher relative abundance of ASVs assigned to *Escherichia-Shigella*. A higher relative abundance of *E. coli* has also been detected in the gut microbiome of pwCF (7, 8, 12, 13, 19, 21). Furthermore, a previous work in a gnotobiotic mouse model demonstrated that colonization with enterohemorrhagic *E. coli* is inhibited by pre-colonization with *B. fragilis* or *Bacteroides vulgatus* (43). In a separate study, *E. coli* O157:H7 was shown to be inhibited by small organic acids (44), including those produced by *Bacteroides* spp. Finally, germ-free CF mice colonized with healthy mouse gastrointestinal (GI) microbiota had higher *Escherichia* GI abundances compared with non-CF germ-free mice (45). Collectively, these results raise the intriguing possibility that the loss of *Bacteroides* and perhaps the propionate it produces, might have implications for interspecies interactions in the context of the microbiota of infants and children with CF, specifically by allowing the expansion of *Escherichia-Shigella*.

We did not detect any differences in the levels of calprotectin, a frequently used marker of intestinal inflammation, in the stool from mice ±*Bacteroides*. While studies have shown that gut microbiome composition is associated with lung health outcomes, evidence is mixed as to whether intestinal calprotectin is correlated with systemic outcomes (17). The results reported here support the idea that while calprotectin may be a useful marker for local inflammation, it may not reflect systemic inflammation or gut-lung axis dynamics in mice.

Given the importance of propionate production by *Bacteroides* in the downregulation of IL-8 in Caco-2 epithelial cells, we hypothesized that the production of propionate by *Bacteroides* would also be required for the downregulation of pro-inflammatory cytokines in *Cftr^F508del^* mice. We designed a similar mouse study to that described above, wherein mice were gavaged with *Bacteroides* that produce propionate or a *Bacteroides* mutant that no longer produced this SCFA using a previously reported Δ*prp* mutant (33). We found that mice gavaged with *Bacteroides* that do not produce propionate had higher *systemic* and *pulmonary* levels of KC (CXCL-1, IL-8 homolog), a pro-inflammatory cytokine. We did not find a significant difference in other cytokines identified as significantly different in the mouse experiment between ±*Bacteroides* treatment groups described above. This finding may be because other *Bacteroides-*secreted metabolites are responsible for the regulation of these other inflammatory pathways, or alternatively, the fact that we used a pool of three clinical strains for the study shown in Fig. 5 and a single lab strain for the study in Fig. 6. Identifying *Bacteroides* strains most effective at colonization and reduction of inflammation will be a key goal going forward.

Finally, no differences in measured propionate levels were observed in stool when using the WT or the Δ*prp* mutant. This finding may not be surprising given that propionate was produced at a detectable level even in the absence of added *Bacteroides*, as well as the high volatility and absorption of SCFAs in the gut. As noted above, the production of SCFA at the right time and place, by a particular microbe, may play a larger role than the bulk levels of these molecules. We also noted that there was no difference in absolute abundance in the Wt versus Δ*prp* mutant in this study, although we see shifts in relative abundance of the Δ*prp* mutant at later time points. The shift in relative abundance of this mutant could reflect its inability to shift the microbiota in the gut to the same extent as the WT strain. As we note above, introduction of *Bacteroides* into the mouse resulted in a reduction of proteobacteria; perhaps this shift is due in part to the production of propionate. Finally, it is also important to consider that *Bacteroides* is functioning in the context of a complex microbial community, which is both producing and consuming SCFA (46, 47)—thus, the elimination of any one genus, even a high abundance one like *Bacteroides*, may not result in a change in the bulk levels of SFCA. While our approach included experiments that are reductionist in nature and help to address a particular molecular mechanism, they should also be considered in this broader, complex community context.

This study highlights the potential for supplementing intestinal *Bacteroides* (i.e., as a probiotic) to modulate systemic and/or pulmonary inflammation. We also highlight the importance of microbially derived propionate and its role in host immune modulation. Finally, we show that microbiome studies in *Cftr^F508del^* mice have the potential to better elucidate the underlying mechanisms in correlative, clinical microbiome studies.

## MATERIALS AND METHODS

### Cell lines

Maintenance of cell lines, bacterial growth, supernatant preparation, and coculture assays were performed as previously described (5), with minor modifications. Details associated with this manuscript are available in the supplemental methods.

### Viability assay

To assess the viability of the CFTR-/- Caco-2 cells under the various treatment conditions assayed, we used an XTT viability assay. XTT was prepared fresh prior to each experiment; 0.5 mg/mL XTT was dissolved in PBS++ (Dulbecco’s PBS + 1 mM MgCl_2_ + 0.1 mM CaCl_2_, pH 8.2) for at least 30 minutes at 55°C. After cooling to room temperature, 0.025 mM menadione was added. After supernatants were removed from the Caco-2 intestinal epithelial cells, the cells were washed 1× with PBS++, and then, 250 μL (24-well plates) or 100 μL (96-well plates) XTT was added to each well. Plates were incubated at 37°C 5% CO_2_ for at least 3 hours or until an orange color developed. One hundred microliters was transferred from each well to a 96-well plate, OD_450_ was measured, and viability was calculated as a percentage of the IL-1β-stimulated control.

### Growth assay

Growth assays of *B. thetaiotaomicron* WT, *B. thetaiotaomicron* Δ*tdk*, and *B. thetaiotaomicron* Δ*tdk*Δ*prp* were completed in brain heart infusion medium (BHIS) as previously described (48) with minor changes. Strains were streaked out onto blood agar + 100 μg/mL gentamicin. Three colonies were then used to inoculate 5 mL and incubated overnight in anaerobic conditions. The overnight cultures were then washed and diluted in BHIS at an optical density (OD) of 0.01 and allowed to incubate in a Cerillo plate reader in anaerobic conditions at 37°C for 26 hours, while collecting OD_600_.

### Fractionation and proteinase K treatment

Supernatants were prepared as described in the supplemental methods from *B. dorei* isolate CFPLTA003_2B. Proteinase K (20 mg/mL, VWR #E195) was added to the supernatant at 2.5 μL/mL, the tube was sealed, and then the samples were incubated at 37°C for 1–2 hours and then at 95°C for 15 minutes to inactivate proteinase K. A mock sample was incubated at the same temperatures for the same amount of time without proteinase K. For fractionation experiments, 500 μL supernatant was applied to 3-kDa size cut-off columns (Amicon Ultra #UFC5000324). Flowthrough and retentate fractions were collected according to the manufacturer’s instructions. The upper retentate was re-diluted to the original volume in fresh sMEM. After processing, standard coculture assays were performed as described above.

### Coculture assays

The CFTR-/- Caco-2 cells were washed 2× with MEM. *Bacteroides* supernatants were diluted 1:1 with fresh sMEM. As a control, freeze-thawed sMEM was also diluted 1:1 with fresh sMEM. Metabolite coculture assays were performed at the specified concentrations of each metabolite in MEM + L-gln. IL-1alpha was added to each condition at a final concentration of 10 ng/mL to stimulate production of IL-8. 250 μL or 100 μL of sterile supernatants, or 1/2 freeze-thawed and 1/2 fresh sMEM as control were added to CFTR-/- Caco-2 cells in 24- or 96-well plates, respectively. After incubation for 6 or 24 hours, as indicated in each experiment, supernatants were removed and centrifuged at 14,000 × *g* for 3 minutes at 4°C. Supernatant from the coculture experiment with CFTR-/- Caco-2 cells was transferred to a clean plastic 96-well plate and stored at −80°C; IL-8 was subsequently quantified by ELISA (BioLegend), as reported (5).

### Metabolite quantification

Undiluted *Bacteroides* supernatants were stored frozen at −80°C prior to metabolite quantification. Frozen samples were shipped to Metabolon for analysis by ultra high-performance liquid chromatography/tandem accurate mass spectrometry (UHPLC/MS/MS) (Metabolon Inc.; Durham, NC). Data were re-scaled to a median of 1, and undetected metabolites were set to the minimum. All statistical analyses were performed on scaled data. SCFAs were quantified by gas chromatography-mass spectrometry (Michigan State University Mass Spectrometry and Metabolomics Core; protocol id MSU_MSMC_010). SCFA concentrations were calculated by normalization to standards. In stool, whole stool pellets were collected from each mouse, weighed, and immediately frozen at −80°C. Two metal beads and 600 μL of methanol were added to each pellet and vortexed for 5 minutes. After a brief centrifugation step, 150 μL of extract was analyzed by gas chromatography-mass spectrometry (Michigan State University Mass Spectrometry and Metabolomics Core; protocol id MSU_MSMC_010). Results were normalized to sample weight at collection. In serum, 12 SCFAs (C2–C8) were analyzed in serum by LC-MS/MS on a Waters Xevo TQ-S mass spectrometer at the Duke proteomics and metabolomics shared resource center. Data analysis was performed with Skyline software (www.skyline.ms). See supplemental methods for additional details.

### 16S rRNA sequencing

DNA was extracted from mouse stool with the Zymo Quick-DNA Fecal/Soil Microbe Miniprep Kit (Cat #D6010), and paired-end amplicon sequencing of the V4–V5 hypervariable region of the 16S rRNA gene was generated with Earth Microbiome Amplicon Libraries (forward primer F515, reverse primer R926) at the UNH Hubbard Center for Genome Studies (Durham, NH) with an Illumina HiSeq 2500 instrument. Stool samples from the Δ*prp* mouse experiment were processed using the Zymo Quick-DNA Fecal/Soil Microbe Miniprep Kit, and sequencing was completed through the Microbial Genome Sequencing Center (MiGS). 2 × 276-bp paired-end amplicon sequences of the V3–V4 and V7–V9 were generated on a V3 MiSeq 600cyc flowcell. The code is available at https://github.com/GeiselBiofilm, and additional details are available in the supplemental methods.

### Absolute abundance quantification

Stool samples collected 13 days after mice were gavaged with strains *B. thetaiotaomicron* Δ*tdk* or *B. thetaiotaomicron* Δ*tdk*Δ*prp* were selected for absolute abundance quantification. Twenty microliters of ZymoBIOMICS Spike-in Control I was added to 10 mg of each stool sample prior to DNA extraction by the Zymo Quick-DNA Fecal/Soil Microbe Miniprep Kit. Extracted DNA was sent for 16-s rRNA sequencing as described above and analyzed for absolute abundance as described in the ZymoBIOMICS Spike-in Control 1 protocol. Absolute abundance was also determined by qPCR as previously described (49), using *B. thetaiotaomicron* VPI-5482-specific probes.

### Whole genome sequencing

Genomes were sequenced at the UNH Hubbard Center for Genome Studies (Durham, NH) with an Illumina HiSeq 2500 instrument. Whole genome sequence information is available under BioProject accession PRJNA557692. Additional details are available in the supplemental methods.

### Detection of candidate *Bacteroides fragilis* toxin gene

To determine whether any *B. fragilis* isolates encoded a gene for the *B. fragilis* enterotoxin (BFT), we first retrieved the Bft amino acid sequence from UniProt (Q6UCA5). We then searched all completed *B. fragilis* genomes on the Integrated Microbial Genomes and Microbiomes system to identify homologs to the UniProt Bft sequence (50). Five genes were identified (Gene IDs: 2649856759, 2649860343, 2649856758, 2848002094, and 2649860342) across the eight organisms searched, and amino acid sequences were downloaded for each gene. An align sequences tblastn search was used to search for the similarity of each amino acid sequence identified above against each *B. fragilis* genome with completed whole genome sequencing (PRJNA557692) in this study.

### Mouse studies

C57BL/6 *Cftr^F508del^* mice [also called *Cftr^em1Cwr^* (34)] deficient in production of the CFTR protein were obtained from Case Western Reserve (34). Mice were fed with ScottPharma LabDiet 5V75 ad libitum and on a 12/12 light cycle. Mice were housed in pairs (within same treatment condition only) where possible or individually. The two mice excluded from the analysis were co-housed with no other mice. Mice were treated with antibiotics for 21 days as previously described to suppress the endogenous intestinal microflora (51). Briefly, antibiotics were prepared at 1 mg/mL in pre-sterilized drinking water. Two antibiotic cocktails were administered over three 7-day periods with two rest days of sterile water provided in between and after the end of antibiotic administration. Ampicillin, cefoprerazone sodium salt, and clindamycin hydrochloride were administered for the first and third 7-day period. Ertapenem, neomycin sulfate, and vancomycin hydrochloride were administered for the second 7-day period. After antibiotic treatment, mice were orally gavaged with 10 μL stool pool from children with CF per gram of mouse weight. Stool pools contained either PBS or spiked-in *Bacteroides* from strains CFPLTA003_2B, SMC7758, and AD126T 3B. Each strain was grown anaerobically for 48 hours as a lawn on 10 blood agar plates and collected fresh for the addition to stool pools. Collected *Bacteroides* were washed and re-suspended in PBS, and an equal volume of PBS only was added to the (−) *Bacteroides* stool pool. The (+) *Bacteroides* condition contained a total inoculum of ~10^9^–10^10^ CFU/mL *Bacteroides* and the (−) Bacteroides condition contained ~10^4^ CFU/mL. Stool was regularly collected from mice before antibiotic treatment and for 2 weeks after oral gavage for analysis of microbiome composition (16S amplicon sequencing, UNH), cytokines (Luminex 32-plex), IgA (ELISA), and SCFAs (GC-MS, Michigan State University). Mice were then anesthetized with isoflurane and oropharyngeally challenged with 0.2 mg/kg of LPS from *P. aeruginosa* (Sigma). Mice were sacrificed with Euthasol 24 hours post-challenge. At sacrifice, whole lungs and intestinal tissue from the cecum, ileum, and colon were collected and immediately fixed in 10% buffered formalin. Whole blood was collected at the time of sacrifice. Serum was separated by allowing samples to stand at room temperature for 1–2 hours and centrifuged at 1,500 × *g* 4°C for 10 minutes. The serum (upper) fraction was removed and stored at −80°C prior to analysis. An ethics statement is available in the supplemental methods.

Similar methods were used for the mouse experiment with the *Bacteroides Δprp* mutant. For this experiment, strains *B. thetaiotaomicron* Δ*tdk* or *B. thetaiotaomicron* Δ*prp* were gavaged into 8 mice, respectively (4m/4f; Table S4). At sacrifice, serum was separated as described above. Lungs were immediately suspended individually after dissection into 5 mL of tissue lysis buffer (11 mg collagenase IV, 2.5 μL DNase I, 250 μL fetal bovine serum (FBS), and 4.75 mL PBS). While being suspended in the tissue lysis buffer, lungs were cut into small pieces with sterile serological scissors and then incubated at 37°C for 45 minutes. Twenty microliters of 0.5M EDTA was added, and the sample was vortexed. Lung samples were then centrifuged at 1,500 rpm for 5 minutes in a microfuge. One milliliter of supernatant from each sample was collected into a 1.5 mL Eppendorf tube, and then the Eppendorf tube was centrifuged at 15,000 rpm for 10 minutes at 4°C to pellet debris. Finally, 200 μL of supernatant was aliquoted into a 96-well round bottom plate and stored at −80°C until analysis for cytokine levels. An aliquot of the tissue lysis buffer was also stored to serve as a blank control. Upon sacrifice, intestines were dissected to isolate three regions: the cecum, proximal colon, and distal colon. Proximal and distal colons were isolated by measuring the full length of the large intestine, cutting it in half, and identifying the half closer to the cecum as proximal and the half closer to the rectum as distal. Intestinal contents were isolated by squeezing out the contents into pre-weighed 2-mL Eppendorf tubes to be serially diluted in PBS and plated for CFUs in both aerobic and anaerobic conditions on blood agar supplemented with 100 μL/mL gentamicin. Intestinal tissue then underwent the same tissue lysis protocol as described for lung tissue.

### Statistics

The complete description of the statistical tests used and details of the tests can be found in Table S3 for the figures in the main body of the text and Table S4 for the figures in the supplemental data.

## SUPPLEMENTAL MATERIAL

Supplemental Methods mbio.03144-23-s0001.pdf Supplemental text.

Supplemental Figures mbio.03144-23-s0002.pdf Figures S1 to S17.

Table S1 mbio.03144-23-s0003.xlsx Strains.

Table S2 mbio.03144-23-s0004.xlsx Mouse studies.

Table S3 mbio.03144-23-s0005.xlsx Stats for main text.

Table S4 mbio.03144-23-s0006.xlsx Stats for supplemental material.

ASM does not own the copyrights to Supplemental Material that may be linked to, or accessed through, an article. The authors have granted ASM a non-exclusive, world-wide license to publish the Supplemental Material files. Please contact the corresponding author directly for reuse.
