# Supplementary material for: Intestinal Bacteroides modulates inflammation, systemic cytokines, and microbial ecology via propionate in a mouse model of cystic fibrosis
Source: mBio. 2024 Jan 5;15(2):e03144-23. doi: 10.1128/mbio.03144-23 (PMC10865972; doi:10.1128/mbio.03144-23)
Supplement: Supplemental Methods — Supplemental text. [file mbio.03144-23-s0001.pdf]

Supplemental Methods

Cell Lines. CFTR was inactivated in a Caco-2 intestinal epithelial cell line by CRISPR/Cas9 targeting of Exon 11 (Hao et al., 2020); three such cell lines designated “S1”, “C9”, and “N5”, along with WT lines that were treated with a mock CRISPR/Cas9 protocol and designated “Y15”, “Y4”, and “N14”. These lines were generously provided by Mitch Drumm at Case Western Reserve University. The S1 cells are referred to as “CFTR-/CFTR- Caco-2 cells” in the text, and all experiments were performed in the S1 background unless otherwise noted. This cell line was maintained as previously described (Antosca et al., 2019). Briefly, cells were cultured at 37°C, 5% CO2 in Advanced MEM (Gibco #12492013) supplemented with 2 mM Glutamax (Gibco #35050061), penicillin-streptomycin (Gibco 50,000 U/500 ml #5140122), and 10% fetal bovine serum (FBS; Gibco; # 15140122). At passaging, cells were seeded into 24-well plates (Falcon #353047) at 100,000 cells/well or 96-well plates (Falcon #353072) at 10,000 cells/well. Cells in 96-well plates were grown for 1 week prior to use. Cells in 24-well plates were grown for 2 weeks prior to use.

Bacterial Culture and Supernatant Preparation. *Bacteroides* isolates were regularly cultured in anaerobic boxes with GasPak EZ pouch system (BD Biosciences) on blood agar, which contained tryptic soy agar (BD Biosciences) + 5% sheep’s blood (Northeast Laboratory Services; Waterville NH). Bacterial supernatants were prepared by inoculating *Bacteroides* grown on blood agar into 5mL freshly prepared MEM (Gibco #51200038) supplemented with components from *Bacteroides* minimal medium

(Martens, Chiang, & Gordon, 2008) and growing anaerobically for 24 hours. *Bacteroides* minimal medium supplements added to MEM are 4mM L-cysteine, 5ug/ml hemin chloride, 100μM $MgCl_2$, 1.4μM $FeSO_4{\cdot}7H_2O$, 50μM $CaCl_2$, 1μg/ml vitamin K3 and 5ng/ml vitamin B12. This medium is referred to as supplemented MEM (sMEM) throughout the text. Oxygen was removed from the liquid medium by de-gassing in a Balch tube with 95% N_2/5% CO_2. Cultures were capped, crimped, and grown standing at 37°C for 24 hours. Cultures were prepared in supplemented MEM (sMEM) and grown for 24 hrs – *Bacteroides* grows faster and to a high density in this medium than in MEM. We incubate the cultures in MEM for 48 hrs so that both sets of cultures would reach saturation. The cultures were collected and plated for colony forming units (CFUs) on blood agar. Supernatants were prepared by centrifuging the culture for 10 minutes at 4°C at 2823 x g, then sterile filtered with a 0.22uM filter to remove remaining bacteria. Supernatants were stored at -80°C prior to use in coculture assays or for quantification of metabolites.

16S rRNA Sequencing. Mouse stool samples were stored immediately at -80°C after collection. After sequencing, primer sequences were removed by CUTADAPT (v 2.10) and all subsequent pre-processing steps were performed in R version 3.6.0. Code is available at https://github.com/GeiselBiofilm. Raw forward-end reads were filtered and trimmed with dada2 version 1.16.0. Reads were then denoised, merged, and chimeras removed. Taxonomy was assigned with the Silva v138.1 training set. Subsequent relative abundance, alpha diversity, ordination, and differential abundance analysis was performed utilizing phyloseq v1.32.0, DESeq2 v1.28.1, and vegan v2.5.7. Whole

Genome Sequencing. Stool from children with or without CF was plated on Bile Esculin Agar and incubated anaerobically for 48 hours to isolate *Bacteroides* and *Parabacteroides* strains. Genomic DNA was prepared by phenol-chloroform extraction prior to sequencing. Genomes were annotated and protein amino acid files generated with prokka (Seemann, 2014). For strains without whole genome sequencing, genus and species were determined by Sanger sequencing of the 16S amplicon for each isolate (Forward primer: AGA GTT TGA TCC TGG CTC AG; Reverse primer: ACG GGC GGT GTG TRC). Ethics Statement. Mouse studies were performed in accordance with the Guide for the Care and Use of Laboratory Animals of the National Institutes of Health (NIH). The protocol was reviewed and approved (0002184) by the Institutional Animal Care and Use Committee (IACUC) at Dartmouth College. The Dartmouth College animal program is accredited with the Association for Assessment and Accreditation of Laboratory Animal Care International (AAALAC) under accreditation number D16-00166 and registered with the US Department of Agriculture (USDA), certificate number 12-R0001, and operates in accordance with Animal Welfare Assurance (NIH/PHS) under assurance number D16-00166 (A3259-01). A total of 26 mice (9 females and 17 males) 10-12 weeks of age at the first antibiotic treatment were included in the study, with 13 mice per condition (Supplemental Table 2). The experiment was independently performed 3 times. Animals were matched by age, sex, and body weight to the extent possible. Stool was collected from all mice, but serum was collected only for two of the three experiments (21 mice). Two mice in the (-) *Bacteroides* condition were excluded due to high relative abundance of *Bacteroides* in

the stool, indicating possible cross contamination. The second study used 16 animals as detailed in Supplemental Table 3.

Growth Assays. To evaluate differences in growth rates for the *B. thetaiotaomicron* Δ *tdk* and *B. thetaiotaomicron* Δ*prp* strains, growth assays were performed as previously described (Robitaille et al., 2023). Briefly, strains were struck onto blood agar and grown anaerobically for 48 hours. Colonies were used to inoculate BHIS liquid medium. A total of 4 wells filled with 3 mL of BHIS were inoculated with ~3 colonies of *Bacteroides* and allowed to grow in anaerobic conditions for 48 hours at 37°C, in a box with a Gas pak. Cultures were then collected, spun down and washed twice with PBS, and diluted in 5mL at OD600 = 0.05. These normalized dilutions were then used to fill 12 wells in a 96-well plate with 200 uL of BHIS. The perimeter, empty wells were filled with sterile water to prevent evaporation. The 96-well plate was covered with a Breathe-Easy gas-permeable membrane and growth (OD600) was measured in an anaerobic hood, every 3 minutes in a Stratus plate reader for 26 hrs.

Attachment Assay. To determine whether there is a difference in the ability of *B. thetaiotaomicron* Δ*tdk* and *B. thetaiotaomicron* Δ*prp* to attach to human colonic epithelial cells, we performed attachment assays with Caco-2 Y15 WT cell lines grown in monolayers on 24 well plates, as previously described (Antosca et al., 2019). After 3 weeks culture, cell monolayers were washed twice with MEM to remove antibiotics. *Bacteroides* were normalized to an OD600 of 0.1 and suspended in sMEM, plus 20ul/mL Oxyrase, +/- 1uL 10 ng/ml IL-1β in a total volume of 250uL of medium and

were added to the well of a 12 well plate. The cocultures were incubated at 37°C with 5% CO_2. After 24hrs, three fractions were collected from each well: (1) the top 200uL of sMEM were collected to plate CFUs/mL of *Bacteroides* as the planktonic population, (2) 200uL PBS was added to wash weakly attached *Bacteroides*, and (3) 200uL PBS was added and Caco-2 cells were scraped up to plate CFUs/mL of *Bacteroides* firmly attached to Caco-2 cells. Collected cells were then serially diluted in PBS and plated on blood agar. Plates were incubated in both anaerobic and aerobic (as a contamination control) conditions at 37°C for 48 hrs, and then CFUs were counted.

References.

Antosca, K. M., Chernikova, D. A., Price, C. E., Ruoff, K. L., Li, K., Guill, M. F., . . . Madan, J. C. (2019). Altered stool microbiota of infants with cystic fibrosis shows a reduction in genera associated with immune programming from birth. *J Bacteriol, 201*(16). doi:10.1128/jb.00274-19

Hao, S., Roesch, E. A., Perez, A., Weiner, R. L., Henderson, L. C., Cummings, L., . . . Drumm, M. L. (2020). Inactivation of CFTR by CRISPR/Cas9 alters transcriptional regulation of inflammatory pathways and other networks. *J Cyst Fibros, 19*(1), 34-39. doi:10.1016/j.jcf.2019.05.003

Martens EC, Chiang HC, Gordon JI. Mucosal glycan foraging enhances fitness and transmission of a saccharolytic human gut bacterial symbiont. Cell Host Microbe. 2008 Nov 13;4(5):447-57. doi: 10.1016/j.chom.2008.09.007. PMID: 18996345; PMCID: PMC2605320.

Robitaille S, Simmons EL, Verster AJ, et al. Community composition and the environment modulate the population dynamics of type VI secretion in human gut bacteria. *bioRxiv*. January 2023:2023.02.20.529031. doi:10.1101/2023.02.20.529031

Seemann T. Prokka: rapid prokaryotic genome annotation. Bioinformatics. 2014 Jul 15;30(14):2068-9. doi: 10.1093/bioinformatics/btu153. Epub 2014 Mar 18. PMID: 24642063.
