# Supplementary material for: Intestinal Bacteroides modulates inflammation, systemic cytokines, and microbial ecology via propionate in a mouse model of cystic fibrosis
Source: mBio. 2024 Jan 5;15(2):e03144-23. doi: 10.1128/mbio.03144-23 (PMC10865972; doi:10.1128/mbio.03144-23)
Supplement: Supplemental Figures — Figures S1 to S17. [file mbio.03144-23-s0002.pdf]

Supplemental Figures

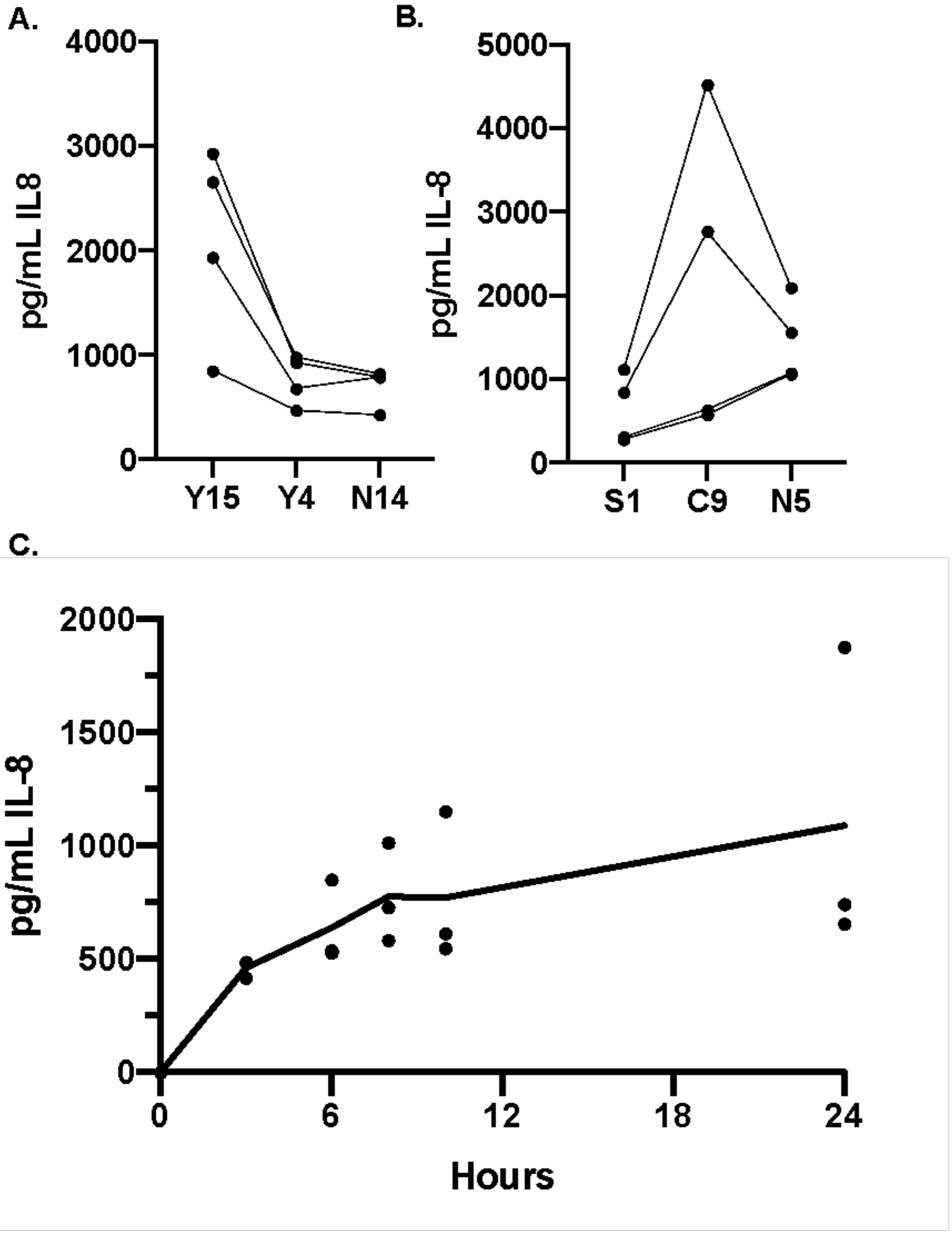

Figure S1. IL-8 production by Caco-2 intestinal epithelial cells stimulated with IL-1β. CFTR-/- Caco-2 intestinal epithelial cell lines were generated by CRISPR/Cas9 editing by Hao et al (Hao et al., 2020). Wild-type cell lines were generated from the same Caco-2 parent as the CFTR-/- lines, but these control lines were subjected to mock CRISPR/Cas9 treatment. Y15, Y4, and N14 are wild-type cell lines, while S1, C9, and N5 are CFTR-/- cell lines. The indicated Caco-2 cell lines were cultured for 2 weeks on plastic in 24-well plates and then treated with 10ng/mL IL-1β in MEM + L-gln medium. IL-8 was quantified by ELISA after 24 hours of IL-1β stimulation for three separate A) wild-type or B) CFTR-/- cell lines. Each point represents the average of four technical replicates from a single biological replicate (n=4), and 3-4 independent biological replicates were performed. Lines connect biological replicates in different cell lines that were performed on the same day. C) IL-8 was quantified by ELISA after 3, 6, 8, 10, and 24 hours of IL-1β exposure to the CFTR-/- S1 Caco-2 cells. Individual points indicate the average of three technical replicates from a single biological replicate (n =3 biological replicates), and the solid line indicates the average of three biological replicates at each time point.

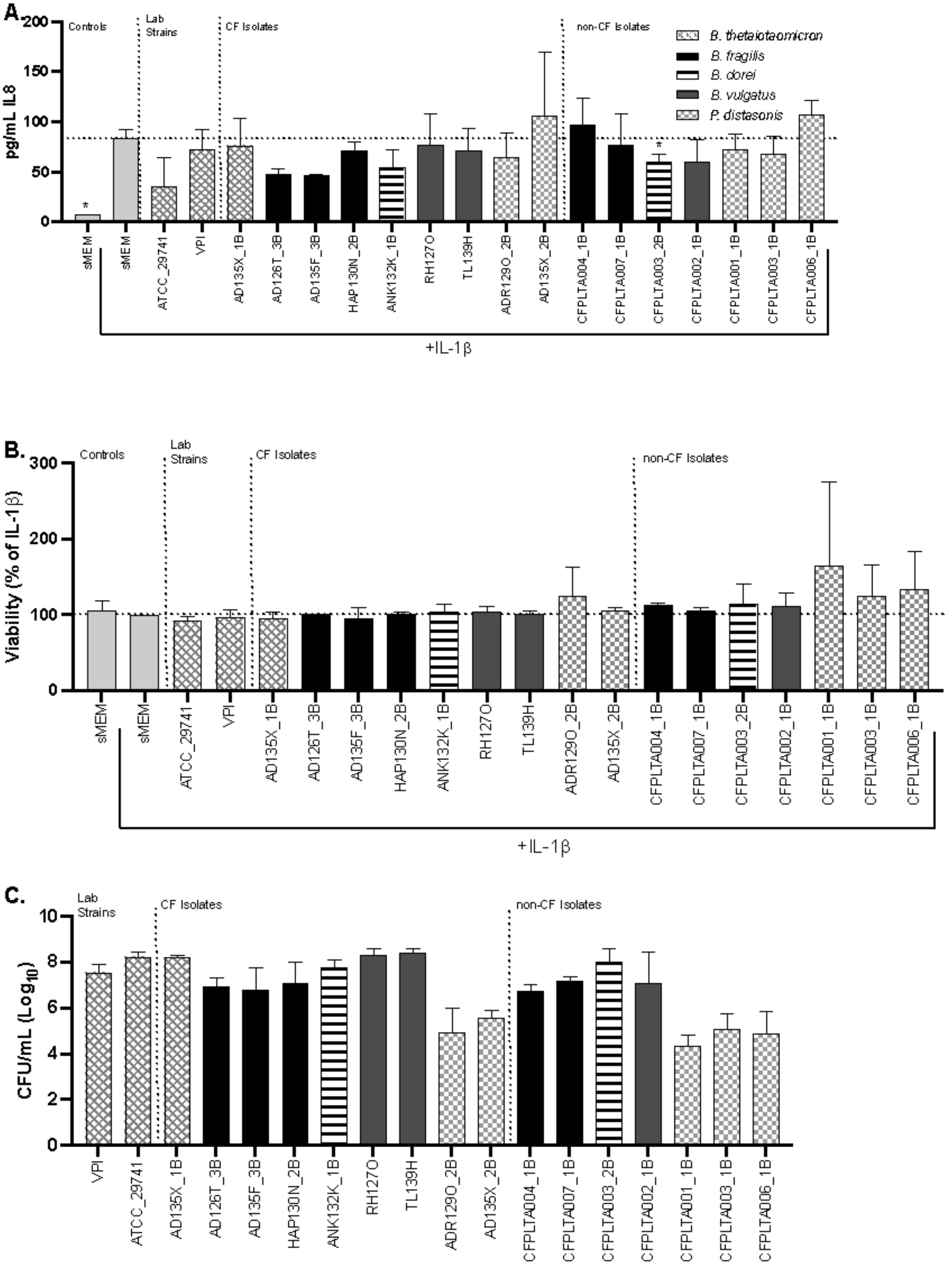

A.
B.
C.
pg/mL IL8
Viability (% of IL-1β)
CFU/mL (Log10)
Controls
Lab Strains
CF Isolates
non-CF Isolates
B. thetaiotaomicron
B. fragilis
B. dorei
B. vulgatus
P. distasonis
sMEM
ATCC_29741
VPI
AD135X_1B
AD126T_3B
AD135F_3B
HAP130N_2B
ANK132K_1B
RH127O
TL139H
ADR129O_2B
AD135X_2B
CFPLTA004_1B
CFPLTA007_1B
CFPLTA003_2B
CFPLTA002_1B
CFPLTA001_1B
CFPLTA003_1B
CFPLTA006_1B
+IL-1β

Figure S2. Order Bacteroidales isolate screen for effects on IL-8. Cell-free, filter sterilized supernatants of the indicated strains were prepared in sMEM, applied to 1-week old CFTR-/- Caco-2 cells grown in 96 well plates, and then incubated for 6 hours as described in the Materials and Methods. Three biological replicates were performed for each isolate. The key in panel A applies to all graphs in the figure. A) IL-8 production was quantified by ELISA for CFTR-/- Caco-2 cells exposed to cell-free, filter sterilized supernatant from each isolate. Significance was tested by paired one-way ANOVA followed by Dunnett's post-test with MEM + IL-1β as the reference. * $p < 0.05$. Strains were not normalized to growth, but growth data is presented in panel C. B) The XTT assay was used to quantify CFTR-/- Caco-2 cellular viability after exposure to the cell-free, filter sterilized supernatant from the indicated isolate. Significance was tested by paired one-way ANOVA followed by Dunnett's post-test with MEM + IL-1β as the reference. C) Bacterial cultures were plated prior to removal of the bacterial cells and filter sterilization to quantify CFU/mL to determine each strain's final growth yield.

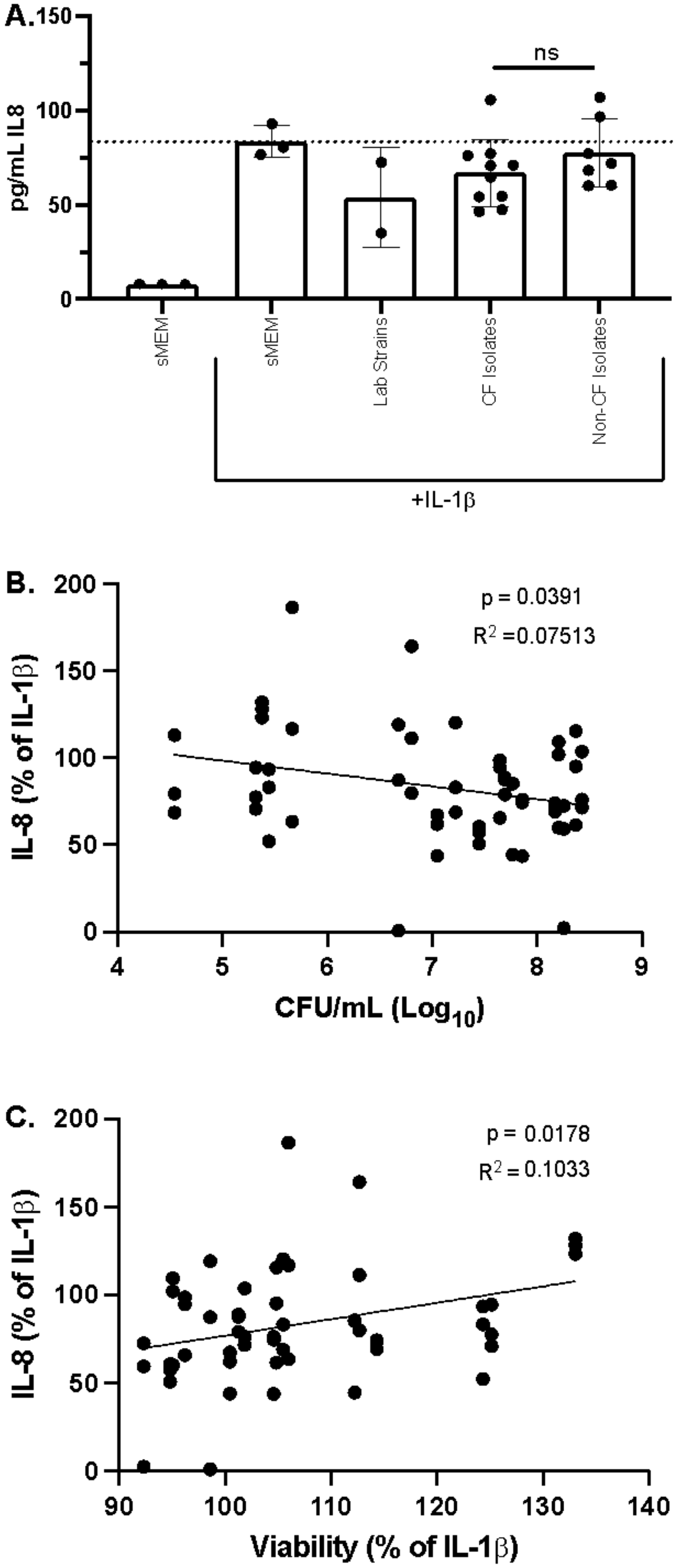
A.
pg/mL IL8
ns
sMEM
sMEM
Lab Strains
CF Isolates
Non-CF Isolates
+IL-1β
B.
IL-8 (% of IL-1β)
p = 0.0391
R2 =0.07513
CFU/mL (Log10)
C.
IL-8 (% of IL-1β)
p = 0.0178
R2 = 0.1033
Viability (% of IL-1β)

Figure S3. Order Bacteroidales isolate correlations with isolate origin, CFU/mL, and cellular viability. Data in this graph are from the isolate screen used to generate the plots in Figure S2. A) Isolates were grouped by origin, and each point represent three biological replicates for a single isolate. An unpaired student's t-test was used to test for significant differences between the CF and non-CF isolates. B) Average Log_{10} CFU/mL of each isolate was plotted versus IL-8 as a percentage of MEM + IL-1β control for each biological replicate. C) Average viability of the cell line in the presence of each isolate was plotted versus IL-8 as a percentage of MEM + IL-1β control for each biological replicate. B-C) Simple linear regression was used to calculate R^2 and p value.

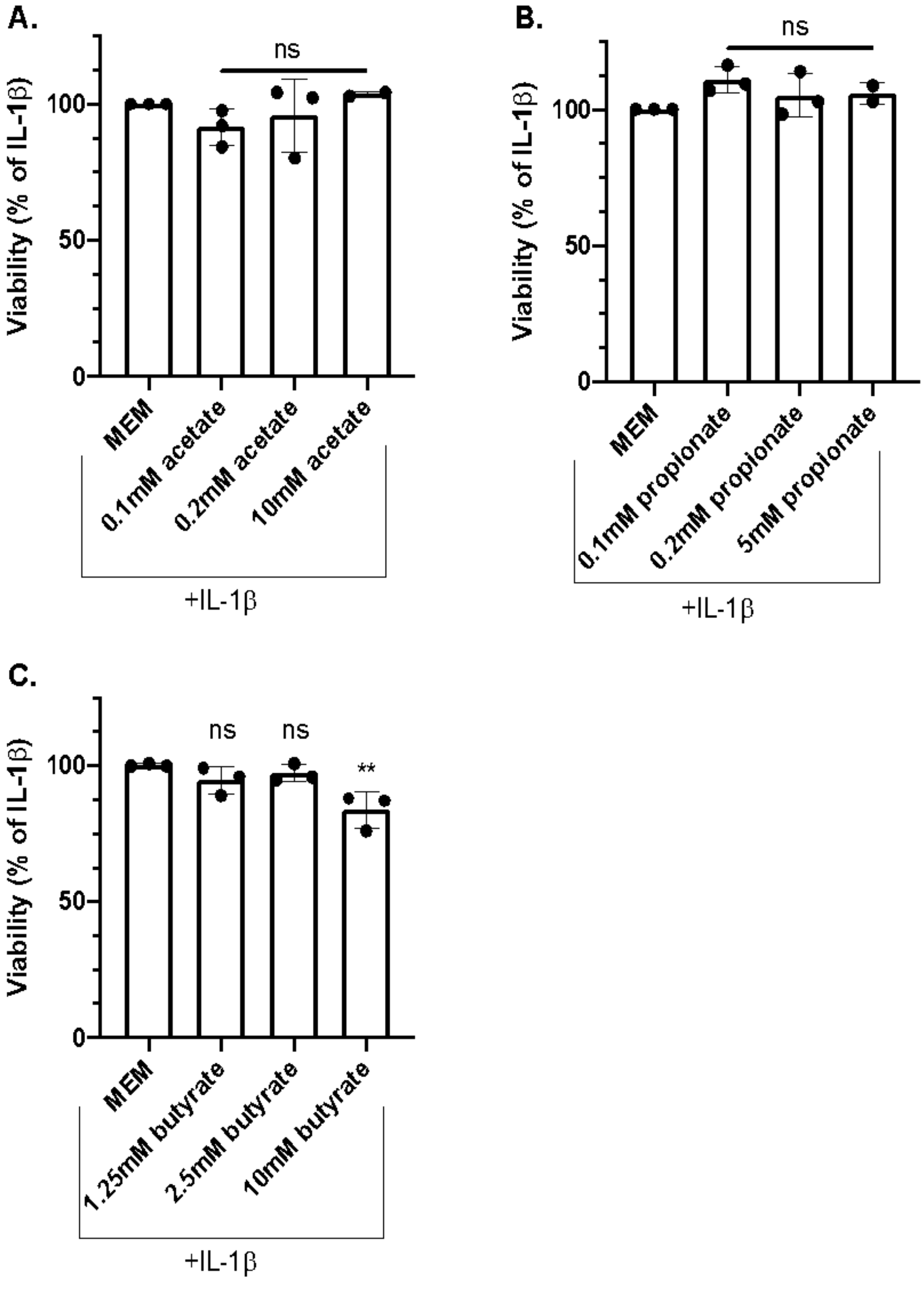
A.
B.
C.
Viability (% of IL-1β)
ns
ns
ns
ns
**
0
50
100
MEM
0.1mM acetate
0.2mM acetate
10mM acetate
0.1mM propionate
0.2mM propionate
5mM propionate
1.25mM butyrate
2.5mM butyrate
10mM butyrate
+IL-1β

Figure S4. Viability of CFTR-/- Caco-2 cell cultured with SCFAs. CFTR-/- Caco-2 cells were cultured for 2 weeks in 24-well plates, and then exposed to MEM with the indicated SCFAs for 24 hrs. Viability was measured by XTT assay after exposure to A) sodium acetate B) sodium propionate and C) sodium butyrate at the indicated concentrations. The value displayed is a percentage of the viability of Caco-2 cells treated with MEM + IL-1β. Significance was tested by unpaired one-way ANOVA with Dunnett's post-test and MEM + IL-1β as the reference. **$p<0.01$.

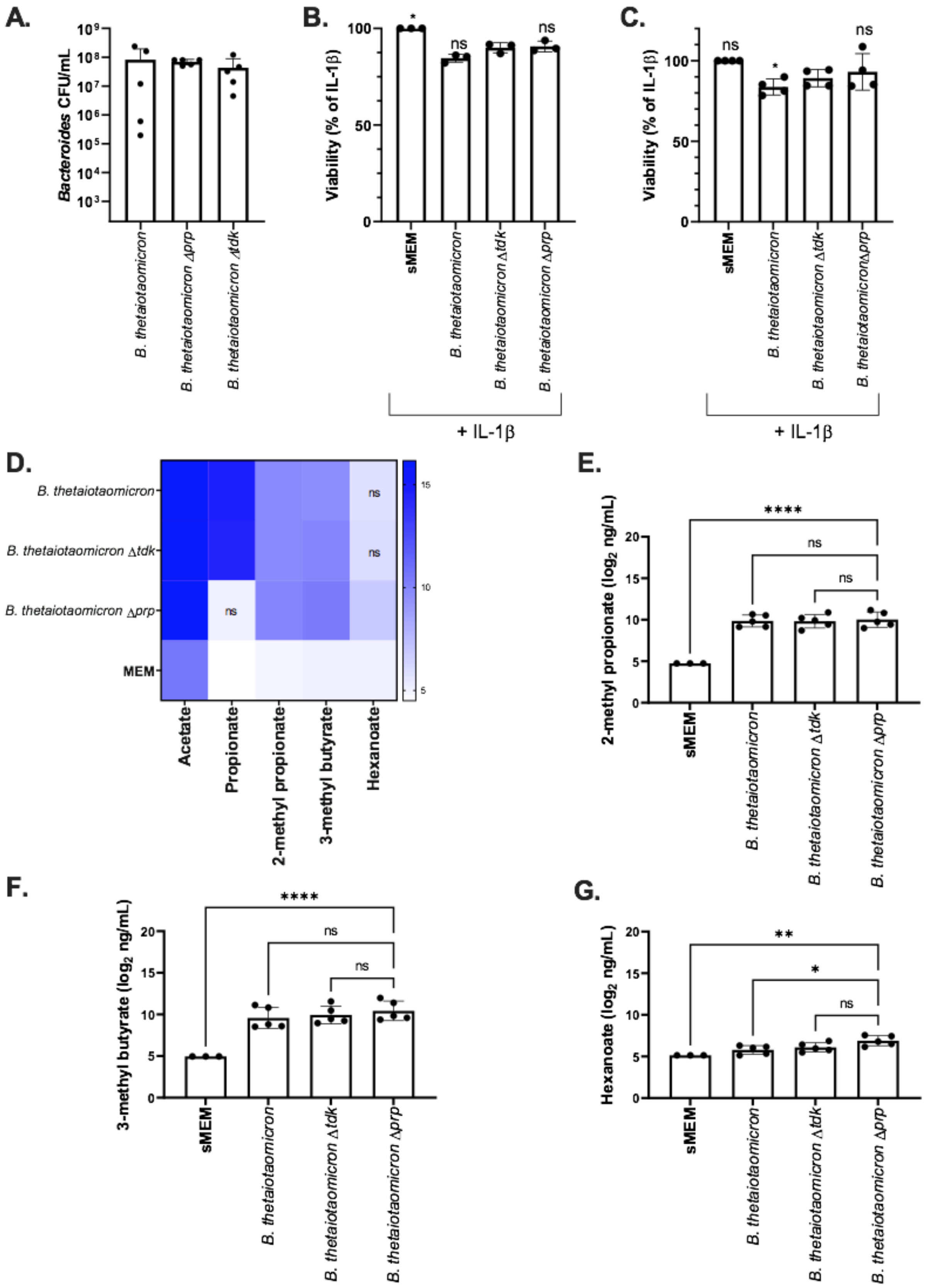
A.
Bacteroides CFU/mL
B. thetaiotaomicron
B. thetaiotaomicron Δprp
B. thetaiotaomicron Δtdk
B.
Viability (% of IL-1β)
sMEM
B. thetaiotaomicron
B. thetaiotaomicron Δtdk
B. thetaiotaomicron Δprp
+ IL-1β
C.
Viability (% of IL-1β)
sMEM
B. thetaiotaomicron
B. thetaiotaomicron Δtdk
B. thetaiotaomicronΔprp
+ IL-1β
D.
B. thetaiotaomicron
B. thetaiotaomicron Δtdk
B. thetaiotaomicron Δprp
MEM
Acetate
Propionate
2-methyl propionate
3-methyl butyrate
Hexanoate
E.
2-methyl propionate (log2 ng/mL)
sMEM
B. thetaiotaomicron
B. thetaiotaomicron Δtdk
B. thetaiotaomicron Δprp
F.
3-methyl butyrate (log2 ng/mL)
sMEM
B. thetaiotaomicron
B. thetaiotaomicron Δtdk
B. thetaiotaomicron Δprp
G.
Hexanoate (log2 ng/mL)
sMEM
B. thetaiotaomicron
B. thetaiotaomicron Δtdk
B. thetaiotaomicron Δprp

Figure S5. ***B. thetaiotaomicron*** **CFU/mL, impact on Caco-2 cell viability, and metabolite production.** A) CFU/mL of cultures prepared after 24 hrs of growth for the indicated isolates. Each point represents a single biological replicate, with 5 total biological replicates performed. No significant differences were identified by one-way ANOVA followed by Tukey's post-test. Viability of the Caco-2 cells was measured by XTT assay after exposure to *B. thetaiotaomicron* supernatants for B) 6 hours and C) 24 hours. Significance was tested by unpaired one-way ANOVA followed by Dunnett's post-test with *B. thetaiotaomicron* Δ*tdk* as the reference. *p<0.05. D-G) SCFAs were quantified (ng/mL) in undiluted, sterile-filtered supernatants by GC-MS. All statistical testing was performed with $\log_2$ transformed concentrations. D) Heatmap of $\log_2$ transformed concentration (ng/mL) of each SCFA. Conditions that are not significantly different from medium control by one-way ANOVA followed by Dunnett's post-test are marked with ns. Pentanoate, 2-methyl pentanoate, and heptanoate were not detected above the LOD. Butyrate was not significantly higher in supernatant than in the medium control. E) 2-methyl propionate and F) 3-methyl butyrate were detected above background but were not significantly different between strains. G) Hexanoate was detected at a small but significantly higher concentration in *B. thetaiotaomicron* Δ*tdk* relative to wild-type *B. thetaiotaomicron*. Values below the limit of detection were thresholded to the LOD. Significance was tested by unpaired one-way ANOVA followed by Dunnett's post-test with *B. thetaiotaomicron* Δ*prp* as the reference; ns = not significant, *p<0.05, **p<0.01, ***p<0.001, ****p<0.0001.

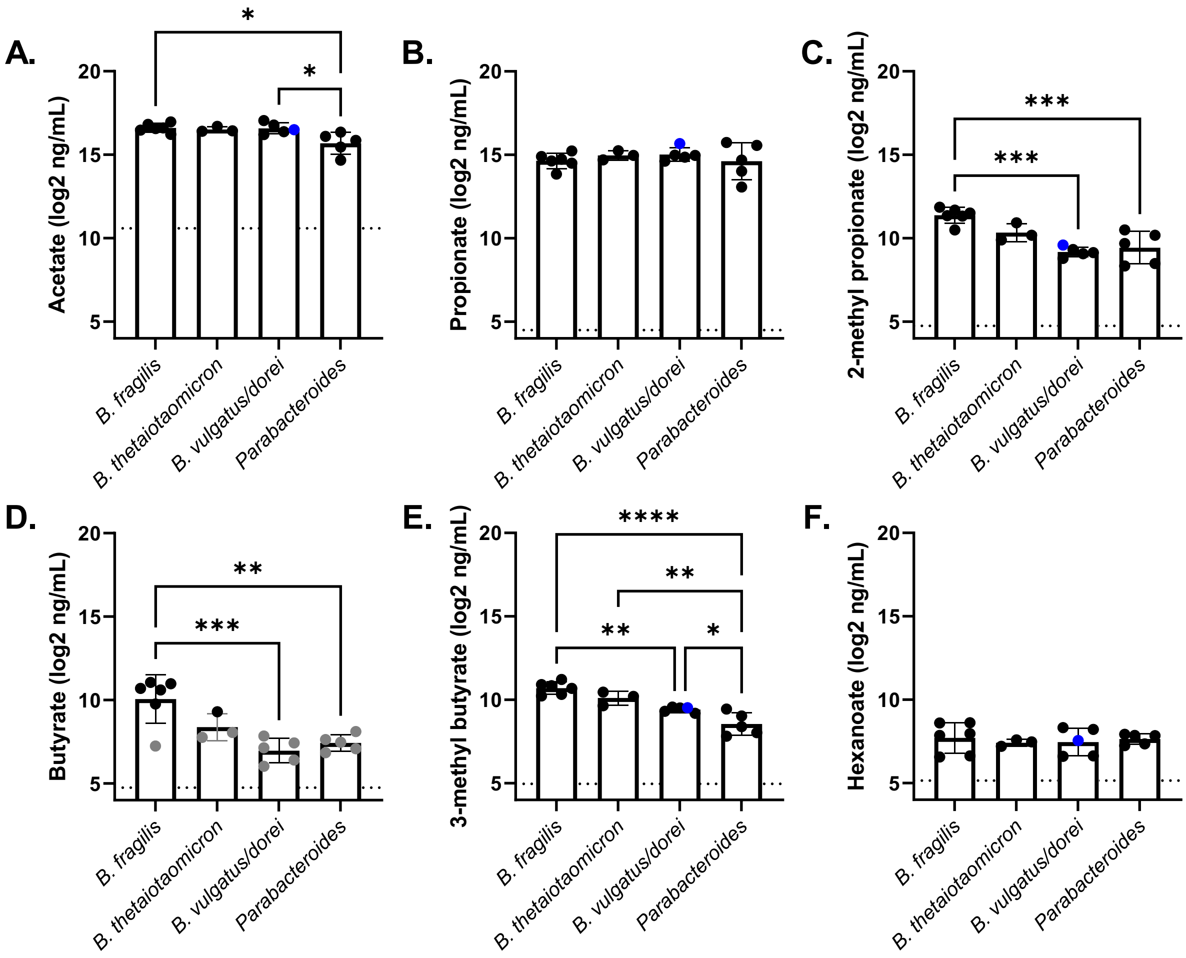

Figure S6. SCFA quantification from isolate supernatants. SCFAs were quantified in undiluted, sterile-filtered supernatants by GC-MS for acetate (A), propionate (B), 2-methyl propionate (C), butyrate (D), 3-methyl propionate (E) and hexanoate (F). Each point indicates the average SCFA concentration in ng/mL from 4-5 biological replicates from an individual isolate. Isolates are grouped in each graph by species. The dashed line indicates the average concentration of each SCFA in medium only. Isolates where a specific SCFA was not significantly higher than the medium control are colored gray. Points representing CFPLTA003_2B are colored blue in each graph, except where the SCFA is not detected significantly above the medium control. Note that only for *B.*

fragilis was the butyrate signal significantly different from the medium control, but this signal did not change in the strains lacking the *buk* or *ptb* genes (not shown), indicating that the signal detected here is likely an artifact or interference. Significance was tested by unpaired one-way ANOVA followed by Tukey's post-test. All significant pairwise differences are annotated; *p<0.05, **p<0.01, ***p<0.001. The black dashed line is the limit of detection.

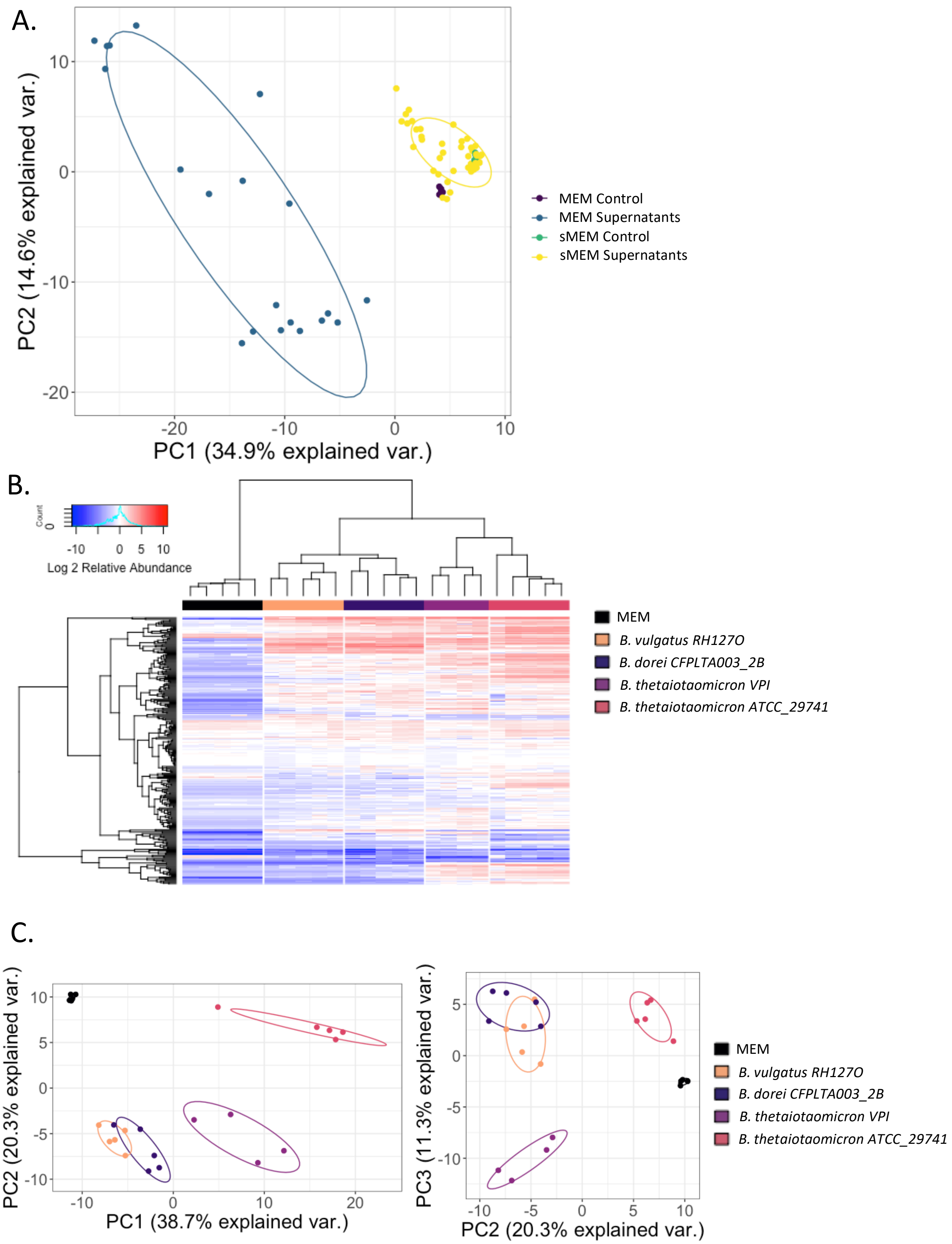
A.
PC2 (14.6% explained var.)
PC1 (34.9% explained var.)
MEM Control
MEM Supernatants
sMEM Control
sMEM Supernatants
B.
Count
Log 2 Relative Abundance
MEM
B. vulgatus RH127O
B. dorei CFPLTA003_2B
B. thetaiotaomicron VPI
B. thetaiotaomicron ATCC_29741
C.
PC2 (20.3% explained var.)
PC1 (38.7% explained var.)
PC3 (11.3% explained var.)
PC2 (20.3% explained var.)
MEM
B. vulgatus RH127O
B. dorei CFPLTA003_2B
B. thetaiotaomicron VPI
B. thetaiotaomicron ATCC_29741

Figure S7. LC-MS quantification of supernatants. Isolates were cultured in MEM + L-gln for 48 hours or sMEM for 24 hours prior to metabolite quantification by LC-MS. A) PCA plot of all metabolites detected in both MEM- and sMEM-grown *Bacteroides* supernatants. Each point represents a single biological replicate and is color-coded by condition. B) Heatmap of all metabolites detected in supernatants from *Bacteroides* cultured in MEM. Each column represents a single biological replicate and is color-coded by strain. The heatmap was generated in R with the Heatmap.2 function. C) PCA plots of all metabolites detected in MEM-grown *Bacteroides* supernatants. Left: PC1 vs PC2. Right: PC2 vs. PC3. Each point represents a single biological replicate and is color-coded by strain. Note: Here, cultures were prepared in supplemented MEM (sMEM) and grown for 24 hrs – *Bacteroides* grows faster and to a high density in this medium than in MEM. We incubate the cultures in MEM for 48 hrs so that both sets of cultures would reach saturation.

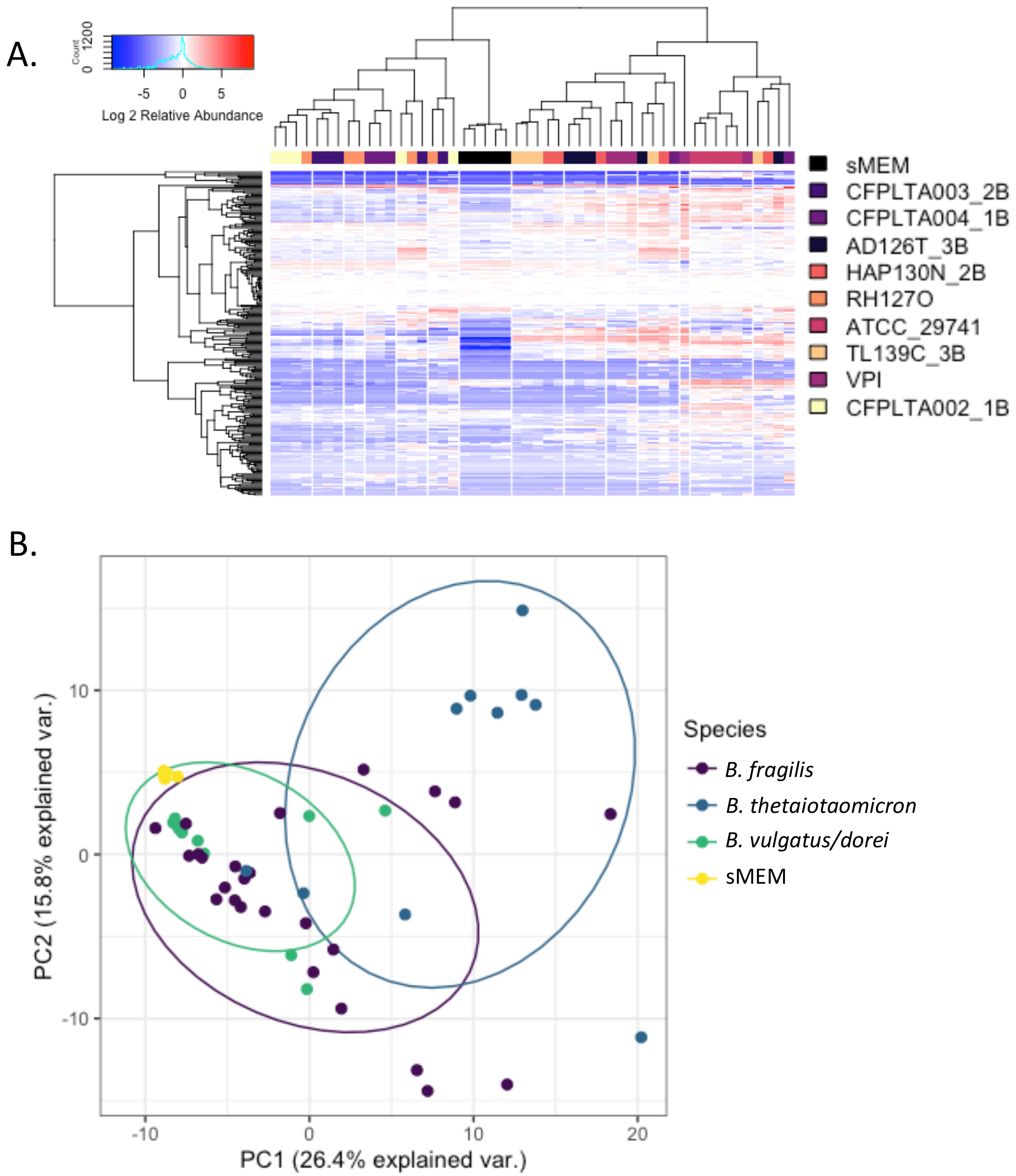

A.
Count
0
1200
-5
0
5
Log 2 Relative Abundance
sMEM
CFPLTA003_2B
CFPLTA004_1B
AD126T_3B
HAP130N_2B
RH127O
ATCC_29741
TL139C_3B
VPI
CFPLTA002_1B
B.
PC2 (15.8% explained var.)
PC1 (26.4% explained var.)
10
0
-10
20
Species
B. fragilis
B. thetaiotaomicron
B. vulgatus/dorei
sMEM

Figure S8. LC-MS quantification of *Bacteroides* and *Parabacteroides* supernatants cultured in sMEM. Isolates were cultured in sMEM for 24 hours prior to metabolite quantification by LC-MS. A) Heatmap of all metabolites detected in supernatants from *Bacteroides* cultured in sMEM. Each column represents a single biological replicate and is color-coded by strain. The heatmap was generated in R with the Heatmap.2 function. B) PCA plots of all metabolites detected in sMEM *Bacteroides* supernatants. Each point represents a single biological replicate and is color-coded by *Bacteroides* species. Note: Here, cultures were prepared in supplemented MEM (sMEM) and grown for 24 hrs – *Bacteroides* grows faster and to a high density in this medium than in MEM cultures, which h are typically incubated for 48 hrs.

.

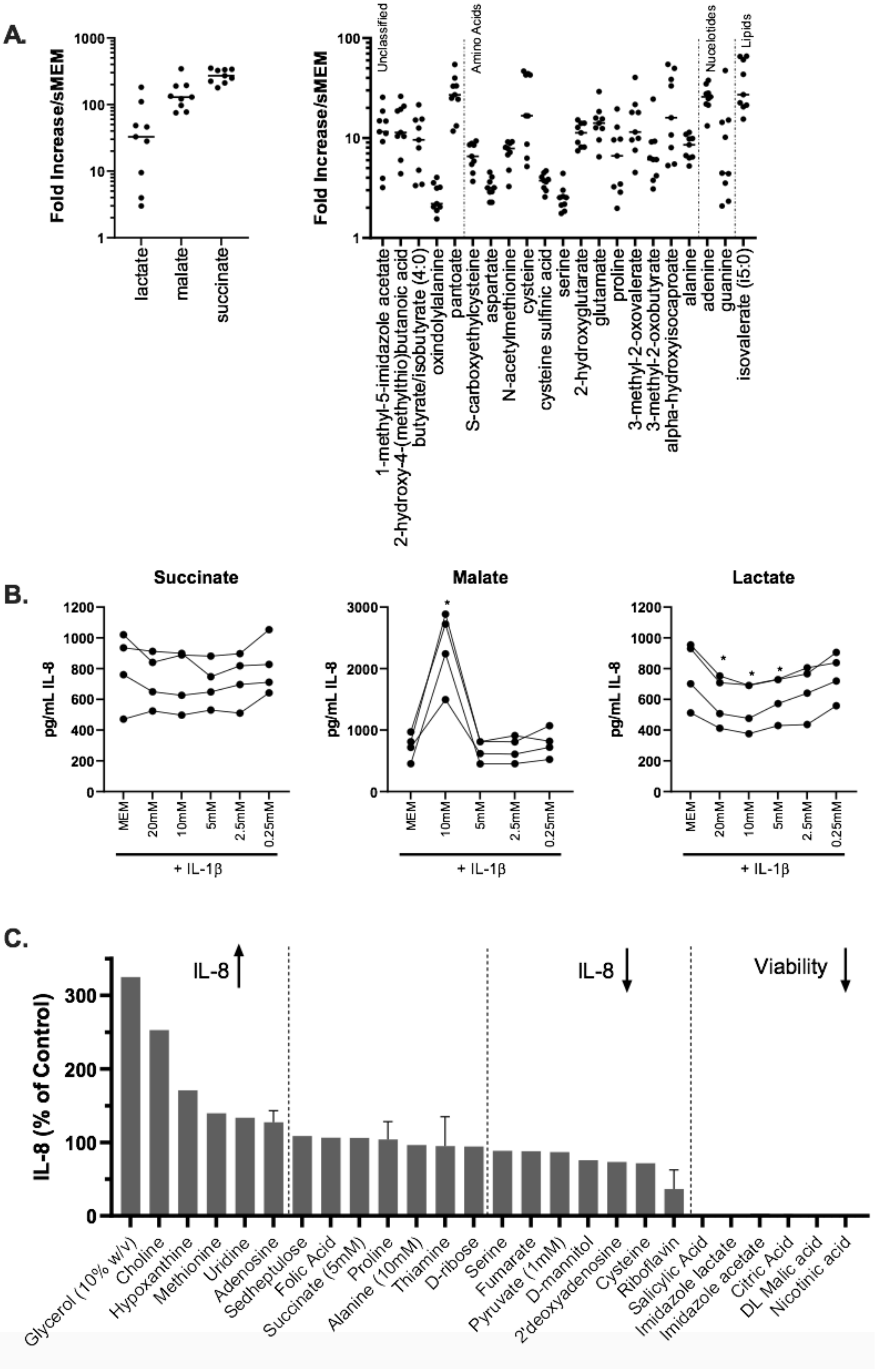
A.
Fold Increase/sMEM
lactate
malate
succinate
Unclassified
Amino Acids
Nucleotides
Lipids
1-methyl-5-imidazole acetate
2-hydroxy-4-(methylthio)butanoic acid
butyrate/isobutyrate (4:0)
oxindolylalanine
pantoate
S-carboxyethylcysteine
aspartate
N-acetylmethionine
cysteine
cysteine sulfinic acid
serine
2-hydroxyglutarate
glutamate
proline
3-methyl-2-oxovalerate
3-methyl-2-oxobutyrate
alpha-hydroxyisocaproate
alanine
adenine
guanine
isovalerate (i5:0)
B.
Succinate
Malate
Lactate
pg/mL IL-8
MEM
20mM
10mM
5mM
2.5mM
0.25mM
+ IL-1β
C.
IL-8 (% of Control)
IL-8
Viability
Glycerol (10% w/v)
Choline
Hypoxanthine
Methionine
Uridine
Adenosine
Sedheptulose
Folic Acid
Succinate (5mM)
Proline
Alanine (10mM)
Thiamine
D-ribose
Serine
Fumarate
Pyruvate (1mM)
D-mannitol
2'deoxyadenosine
Cysteine
Riboflavin
Salicylic Acid
Imidazole lactate
Imidazole acetate
Citric Acid
DL Malic acid
Nicotinic acid

Figure S9. Impacts of highly produced metabolites on IL-8 level. A) LC-MS quantification of metabolites produced by *Bacteroides* and *Parabacteroides* supernatants cultured in sMEM. *Bacteroides* isolates in this panel were cultured in sMEM for 24 hours prior to metabolite quantification by LC-MS. All metabolites detected >3-fold over sMEM control for every isolate and displayed as a ratio of the metabolite detected in supernatant/ sMEM control. The panel on the left highlights SCFAs. B) CFTR-/- Caco-2 cells were cultured for 2 weeks in 24-well plates and then treated with IL-1β alone, or with the addition of the indicated metabolite concentrations. IL-8 was quantified by ELISA after 24 hours of culture. Each point indicates the average of 4 technical replicates from a single biological replicate. Lines connect results from experiments performed on the same day. Significance was tested by unpaired one-way ANOVA followed by Dunnett's post-test with MEM + IL-1β as the reference. *$p<0.05$. C) CFTR-/- Caco-2 cells were cultured for 2 weeks in 24-well plates and then treated with IL-1β alone, or with the addition of 20mM of each metabolite unless otherwise indicated. All compounds were diluted into MEM, the medium used to grow the CFTR-/- Caco-2 cells. IL-8 was quantified by ELISA after 24 hours of incubation and normalized to the MEM + IL-1β control condition.

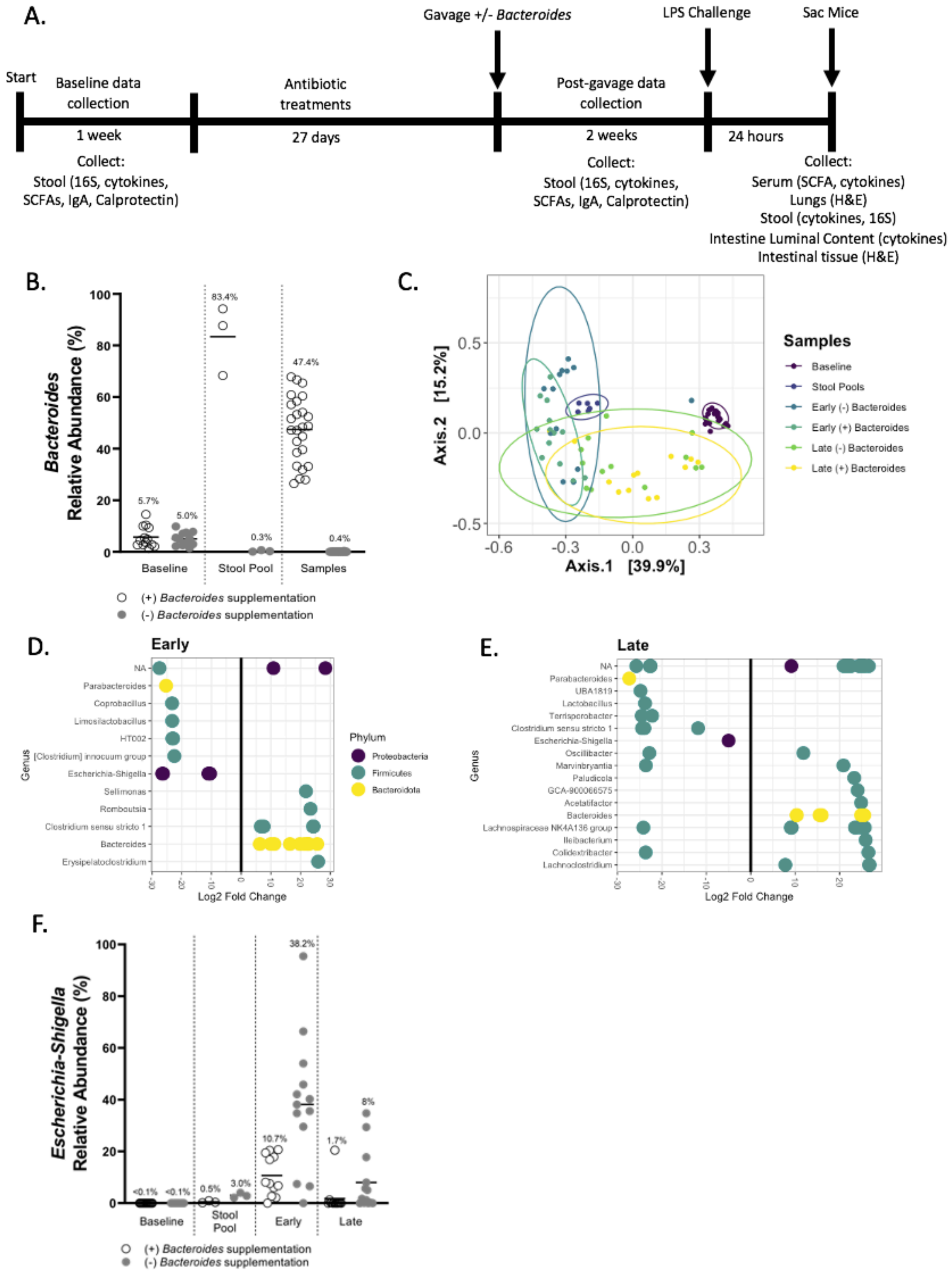
A.
Start
Baseline data collection
1 week
Collect:
Stool (16S, cytokines, SCFAs, IgA, Calprotectin)
Antibiotic treatments
27 days
Gavage +/- Bacteroides
Post-gavage data collection
2 weeks
Collect:
Stool (16S, cytokines, SCFAs, IgA, Calprotectin)
LPS Challenge
24 hours
Sac Mice
Collect:
Serum (SCFA, cytokines)
Lungs (H&E)
Stool (cytokines, 16S)
Intestine Luminal Content (cytokines)
Intestinal tissue (H&E)
B.
Bacteroides Relative Abundance (%)
Baseline
Stool Pool
Samples
5.7%
5.0%
83.4%
0.3%
47.4%
0.4%
(+) Bacteroides supplementation
(-) Bacteroides supplementation
C.
Axis.1 [39.9%]
Axis.2 [15.2%]
Samples
Baseline
Stool Pools
Early (-) Bacteroides
Early (+) Bacteroides
Late (-) Bacteroides
Late (+) Bacteroides
D.
Early
Genus
NA
Parabacteroides
Coprobacillus
Limosilactobacillus
HT002
[Clostridium] innocuum group
Escherichia-Shigella
Sellimonas
Romboutsia
Clostridium sensu stricto 1
Bacteroides
Erysipelatoclostridium
Log2 Fold Change
Phylum
Proteobacteria
Firmicutes
Bacteroidota
E.
Late
Genus
NA
Parabacteroides
UBA1819
Lactobacillus
Terrisporobacter
Clostridium sensu stricto 1
Escherichia-Shigella
Oscillibacter
Marvinbryantia
Paludicola
GCA-900066575
Acetatifactor
Bacteroides
Lachnospiraceae NK4A136 group
Ileibacterium
Colidextribacter
Lachnoclostridium
Log2 Fold Change
F.
Escherichia-Shigella Relative Abundance (%)
Baseline
Stool Pool
Early
Late
<0.1%
<0.1%
0.5%
3.0%
10.7%
38.2%
1.7%
8%
(+) Bacteroides supplementation
(-) Bacteroides supplementation

Figure S10. Mice treated with antibiotics and gavaged with CF stool +/- *Bacteroides*. A) Outline of mouse experimental timeline. B) Relative abundance of *Bacteroides* ASVs in “Stool Pools” (human stool +/- *Bacteroides*, used for gavage) and pooled mouse stool collected “Early” (2-4 days) or “Late” (12-13 days) post-gavage from mice (samples). C) Community structure was analyzed by multidimensional scaling (MDS) ordination based on Bray-Curtis dissimilarity coefficients and significance was analyzed by PERMANOVA. Stool samples are grouped by Early (2-4 days) and Late (12-13 days) post-gavage collection dates. The two *Bacteroides* conditions were significantly different Early ($p = 0.014$) but not Late post-gavage ($p = 0.085$). D-E) Significant alterations in specific ASVs were analyzed by DESeq2 either at the Early (D) or Late (E) time point. The log2 fold change of (+) supplemented *Bacteroides* versus (-) supplemented *Bacteroides* condition is displayed on the X-axis such that values >0 indicate an ASV that is significantly higher in the (+) *Bacteroides* condition. Each point represents an individual ASV and is labeled by genus on the Y-axis and color coded by phylum. F) Relative abundance of *Escherichia-Shigella* ASVs in “Stool Pools” (human stool +/- *Bacteroides*, used for gavage – this is the baseline condition) and mouse stool collected “Early” (2-4 days) or “Late” (13-13 days) post-gavage from mice with the indicated gavage condition.

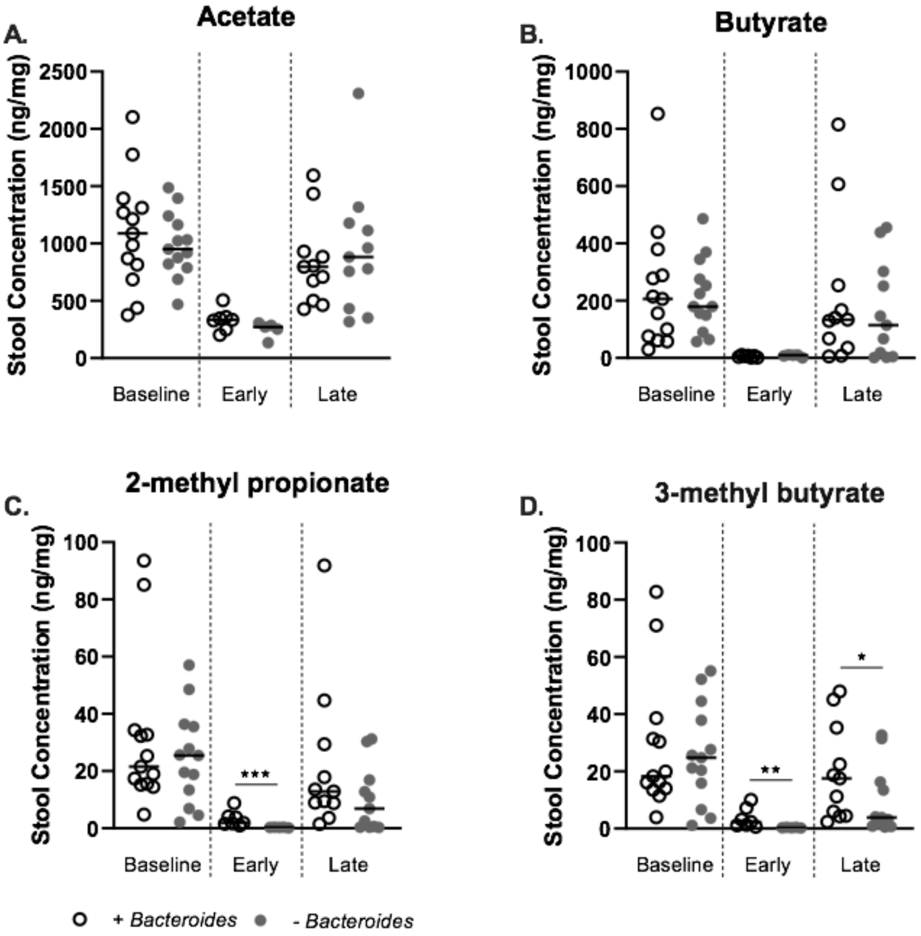

Figure S11. SCFA quantification in mouse stool. SCFAs A) acetate, B) butyrate, C) 2-methyl propionate, and D) 3-methyl butyrate were quantified in mouse stool by GC-MS at baseline, and in the early and late window post-gavage. The bottom left figure legend indicates the condition for all panels. Statistical significance was analyzed by linear model between the (+) *Bacteroides* and (-) *Bacteroides* conditions for each window (early, late) on log2-transformed concentrations, accounting for treatment, experiment, cage, and sex of mouse. All significant results are reported on the graph. ns = not significant. * $p<0.05$, ** $p< 0.01$, ***$p<0.001$.

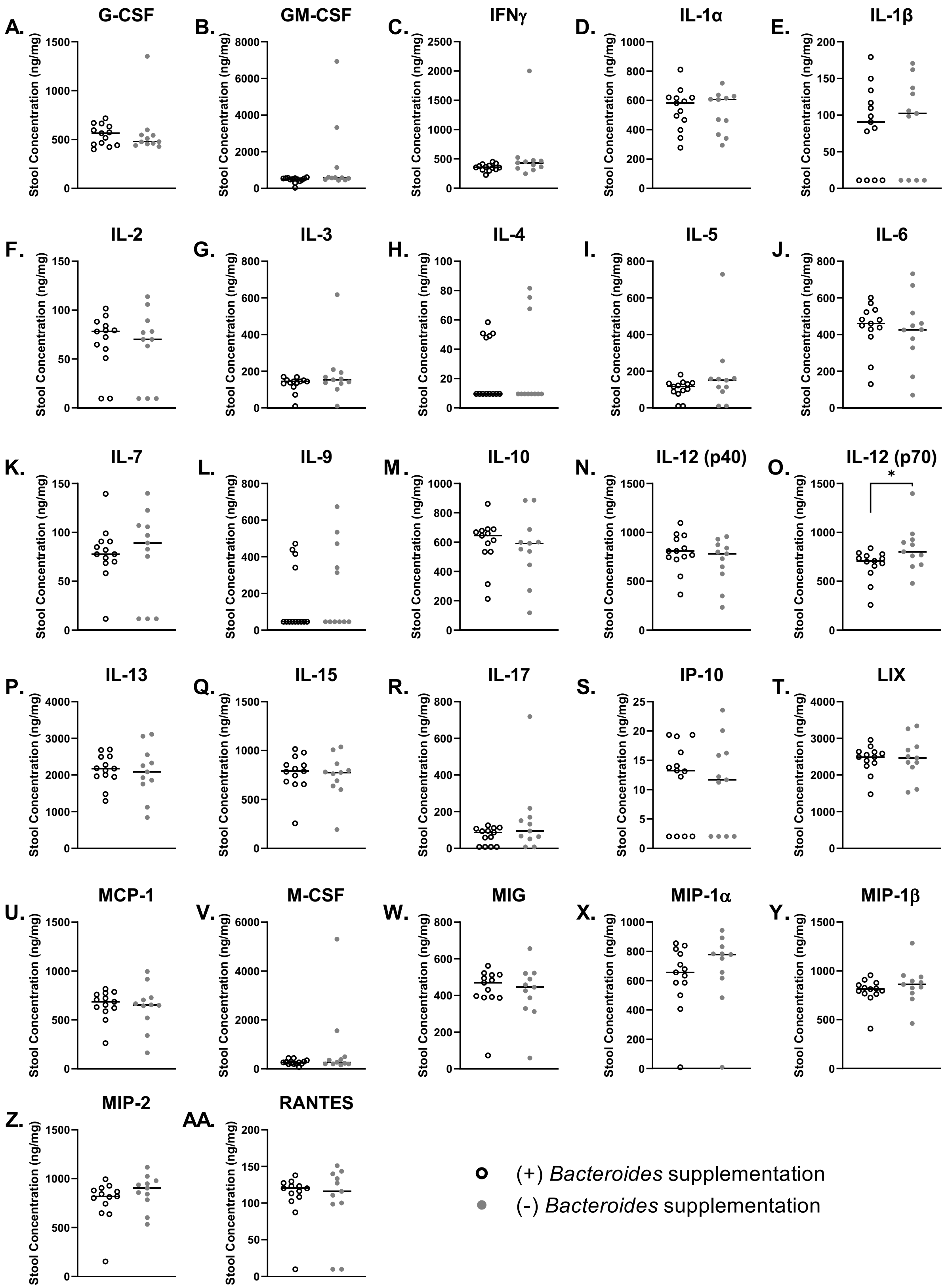

A. G-CSF
B. GM-CSF
C. IFNγ
D. IL-1α
E. IL-1β
F. IL-2
G. IL-3
H. IL-4
I. IL-5
J. IL-6
K. IL-7
L. IL-9
M. IL-10
N. IL-12 (p40)
O. IL-12 (p70)
*
P. IL-13
Q. IL-15
R. IL-17
S. IP-10
T. LIX
U. MCP-1
V. M-CSF
W. MIG
X. MIP-1α
Y. MIP-1β
Z. MIP-2
AA. RANTES
Stool Concentration (ng/mg)
(+) Bacteroides supplementation
(-) Bacteroides supplementation

Figure S12. Cytokines detected in mouse stool. Mouse stool samples from three independent experiments were diluted 1:50 by weight in extraction buffer (Immunodiagnostik KR6936) and further diluted 1:5 in PBS prior to cytokine quantification by Luminex 32-plex. Each cytokine is indicated above the graph in panels A-AA. Each point indicates a single stool sample from a different mouse, and the bar indicates the median of all samples. In the (+) supplemented *Bacteroides* condition n=13, and in the (-) supplemented *Bacteroides* condition n=11. Cytokines for which fewer than 5 of the samples had cytokine within the limit of detection (LOD) (TNFα, Eotaxin, and IP-10) were excluded from the analysis. Samples with values below the limit of detection (LOD) were excluded. Statistical significance was analyzed by linear model on $\log_2$ transformed concentrations, accounting for treatment, experiment, cage, and sex of mouse, and pairwise results are reported in each panel. ns = not significant. The only significant cytokine (before multiple comparisons) is IL-12p70, * $p<0.05$.

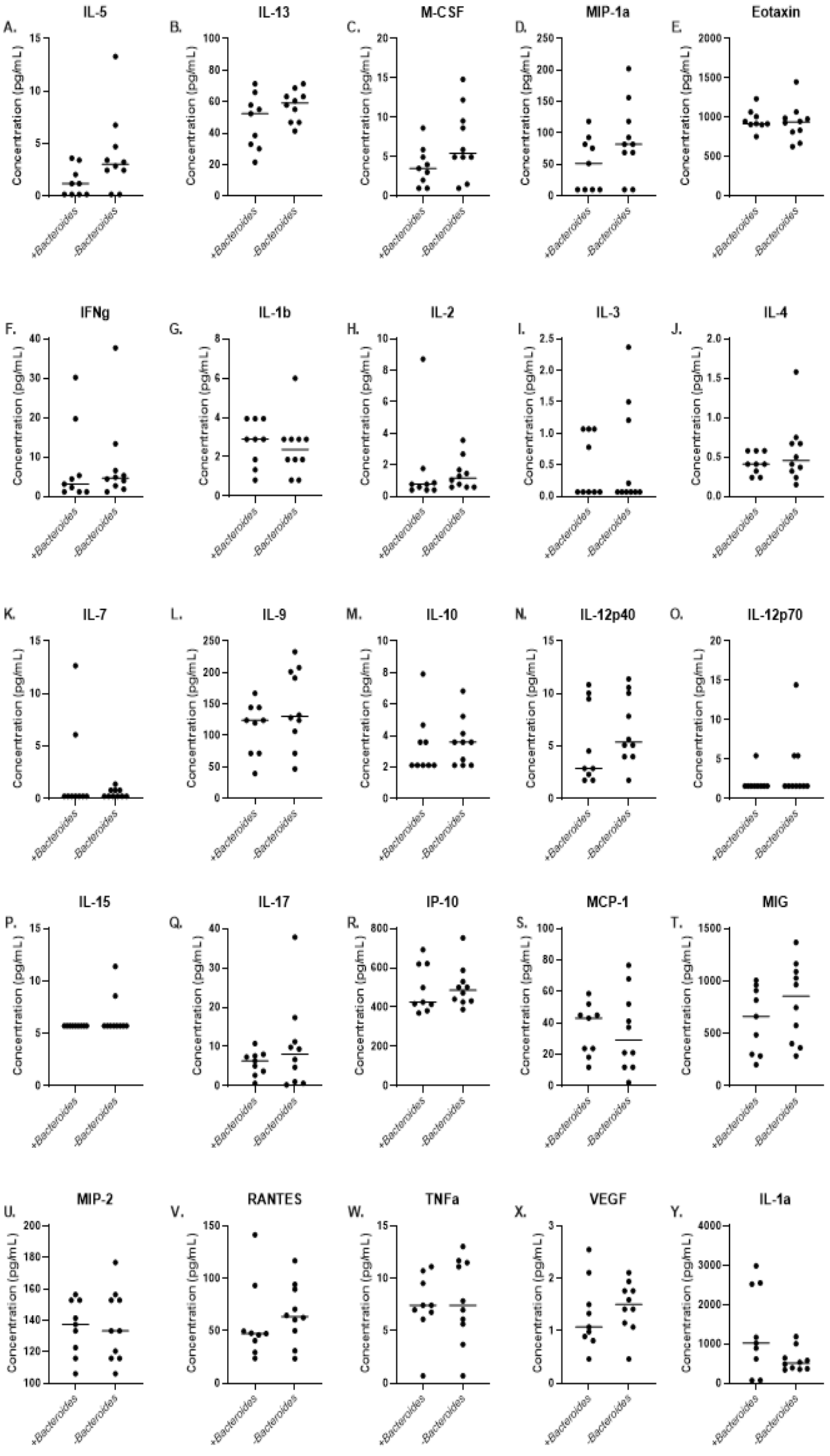
A. IL-5
B. IL-13
C. M-CSF
D. MIP-1a
E. Eotaxin
F. IFNg
G. IL-1b
H. IL-2
I. IL-3
J. IL-4
K. IL-7
L. IL-9
M. IL-10
N. IL-12p40
O. IL-12p70
P. IL-15
Q. IL-17
R. IP-10
S. MCP-1
T. MIG
U. MIP-2
V. RANTES
W. TNFa
X. VEGF
Y. IL-1a
Concentration (pg/mL)
+Bacteroides
-Bacteroides

Figure S13. Cytokines detected in mouse serum. Mouse serum samples collected from two independent experiments were diluted 1:2 in PBS prior to cytokine quantification by Luminex 32-plex. Each cytokine is indicated above the graph in panels A-Y. Each point indicates cytokine quantity in a single serum sample from an individual mouse, and the bar indicates the median of all samples. In the (+) supplemented *Bacteroides* condition n=9, and in the (-) supplemented *Bacteroides* condition n=10. Cytokines where fewer than 5 of the samples had cytokine within the limit of detection (LOD) (GM-CSF, LIF, and LIX) were excluded from the analysis. Samples with values below the LOD were thresholded to the minimum LOD. Statistical significance was analyzed by linear model on log2 transformed values, accounting for treatment, experiment, cage, and sex of mouse. There was no significant difference in the cytokines shown here (see main text for cytokines with significant differences).

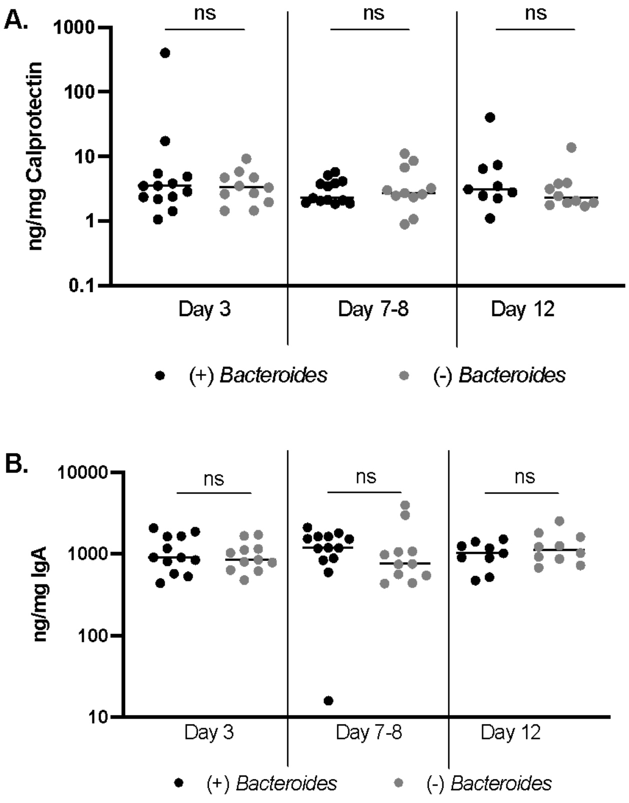

Figure S14. Quantification of stool calprotectin and IgA. Mouse stool samples were diluted 1:50 by weight in extraction buffer (Immunodiagnostik KR6936) and then separately quantified by ELISA for A) calprotectin and B) IgA. Statistical significance was tested by unpaired t-test on $\log_2$ transformed concentrations for the indicated pairwise comparisons. ns = not significant.

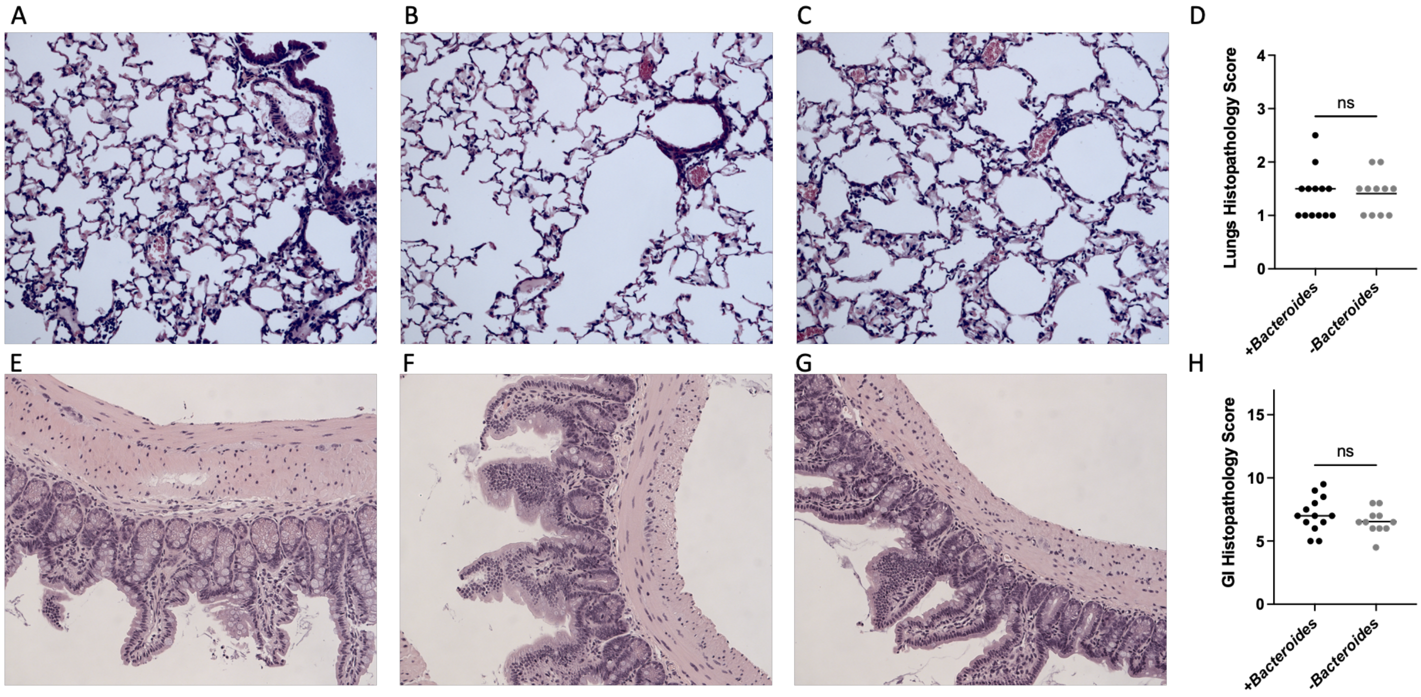
A
B
C
D
E
F
G
H
Lungs Histopathology Score
GI Histopathology Score
ns
+Bacteroides
-Bacteroides
0
1
2
3
4
5
10
15

Figure S15. Analysis of pathology. Lung and intestinal tissue were collected at sacrificing and preserved in 10% buffered formalin. After a minimum of 24 hours of fixation, samples were transferred to 70% ethanol and stained with Hematoxylin and Eosin (H&E) to visualize infiltration of immune cells. A-B) Representative 20X images from lung tissue in the (-) supplemented *Bacteroides* group. C) Representative 20X image from lung tissue in the (+) supplemented *Bacteroides* group. All lung tissue demonstrated mild to no inflammatory response. D) Histopathology score for the lungs showed no significant difference between intestinal *Bacteroides* condition (p=0.287). E-F) Representative 20X images from intestinal tissue in the (-) supplemented *Bacteroides* group. G) Representative image from intestinal tissue in the (+) supplemented *Bacteroides* group. H) Histopathology score for the intestines showed no significant difference between intestinal *Bacteroides* condition (p=0.079). Evaluation of gastrointestinal inflammation was completed independently by two pathologists using blinded data based on previously established scoring rubrics for evaluation of intestinal inflammation in mouse models (Erben et al., 2014). Lung inflammation was evaluated independently by two pathologists using blinded data and scored using a previously established scoring system for lung injury in CF mice (Semaniakou etl. Al, 2020). Scores were averaged and statistical significance was determined using linear model on $\log_2$ transformed average scores of the lungs and not transformed average scores of the intestines, accounting for treatment, experiment, cage, and sex of mouse. ns = not significant.

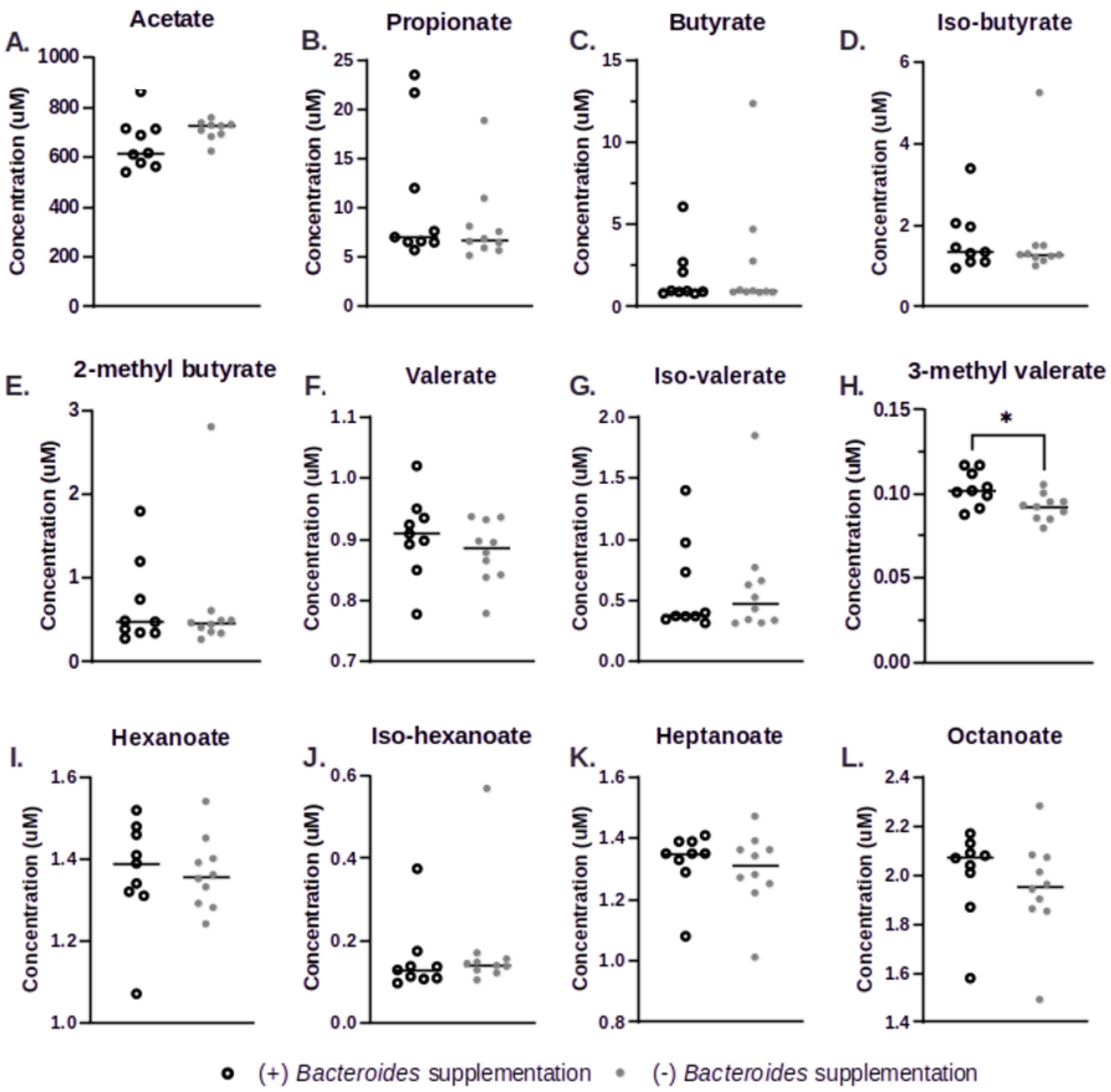

Figure S16. SCFA quantification in mouse serum. SCFAs were quantified in mouse serum by LC-MS/MS (Duke Proteomics and Metabolomics Shared Resource). Each SCFA is indicated above the graph in panels A-L. Statistical significance was analyzed by linear model on log2 transformed concentrations, accounting for treatment, experiment, cage, and sex of mouse. Pairwise results are reported in each panel. * $p<0.05$.

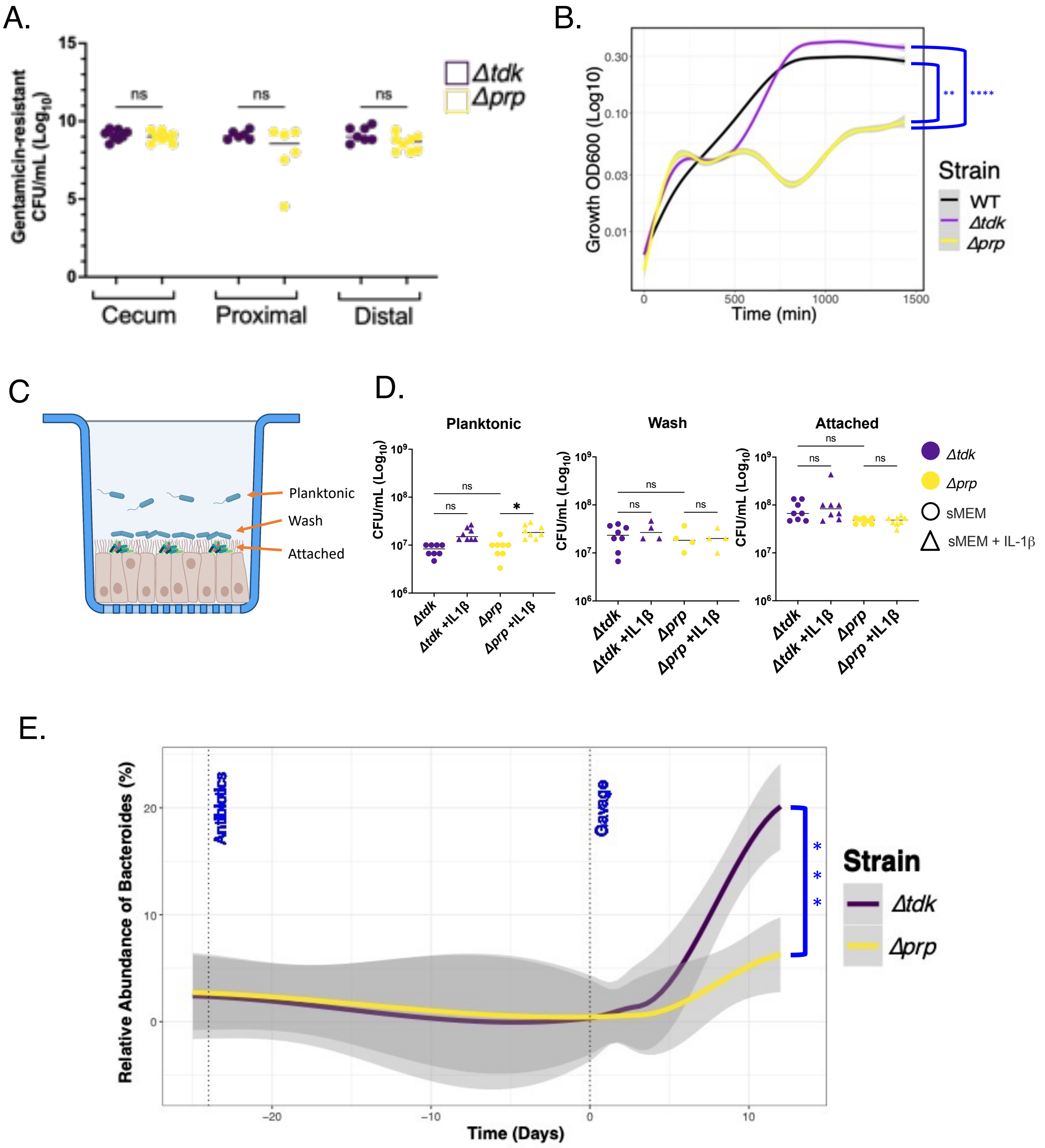

A.
Gentamicin-resistant CFU/mL (Log10)
ns
Δtdk
Δprp
Cecum
Proximal
Distal
B.
Growth OD600 (Log10)
Time (min)
**
****
Strain
WT
Δtdk
Δprp
C
Planktonic
Wash
Attached
D.
Planktonic
Wash
Attached
CFU/mL (Log10)
ns
*
Δtdk
Δtdk +IL1β
Δprp
Δprp +IL1β
sMEM
sMEM + IL-1β
E.
Relative Abundance of Bacteroides (%)
Antibiotics
Gavage
Time (Days)
*
*
*
Strain
Δtdk
Δprp

Figure S17. Analysis of the *Bacteroides* Δ*tdkprp* mutant. A) Mice were gavaged with *Bacteroides* Δ*tdk* (purple) or *Bacteroides* Δ*prp* (yellow). At sacrifice intestines were dissected into three regions: cecum, proximal colon, and distal colon. Intestinal contents for each were serially diluted and plated to count CFU/mL on blood agar +100ug/mL gentamicin in order to select for *Bacteroides*. Each point indicates stool collected from a single mouse. Statistical significance was determined by Kruskal-Wallis with Dunn's multiple pair-wise comparisons test. B) Growth assays were performed in BHIS liquid medium and allowed to grow in anaerobic conditions at 37°C for 26hrs. Strains included were *Bacteroides* VPI WT (black), *Bacteroides* VPI *Δtdk* (purple), and *Bacteroides* VPI *Δprp* (yellow). Statistical significance was determined by Kruskal-Wallis with Dunn's on OD_{600} recorded at 26hrs, along with Dunn's multiple pair-wise comparisons test. **p<0.01. ***p<0.001. C) Schematic depicting three fractions collected from each well in *Bacteroides* attachment assays on human colon epithelial cells. D) Fractions were serially diluted and plated to count CFU/mL on blood agar. Each point indicates a single well from two 24 well plates. Colon cell lines were inoculated with *Bacteroides* Δ*tdk* (purple) or *Bacteroides* Δ*prp* (yellow). *Bacteroides* were added, suspended in sMEM (circle) or sMEM + IL-1β to stimulate inflammatory response by colon cells. Statistical significance was determined by Kruskal-Wallis with Dunn's multiple pair-wise comparisons test. *p<0.05. **p<0.01. ***p<0.001. E) Stool samples were profiled for microbial composition (relative abundance) through 16S rRNA gene amplicon sequencing to determine *Bacteroides* relative abundance across the experiment timeline. Ribbon plots depicting the relative abundance of *Bacteroides* in mice gavaged with *Bacteroides* Δ*tdk* (purple) or *Bacteroides* Δ*tdk* Δ*prp* (yellow), where dotted lines indicate start of antibiotic treatment, then gavage of CF Stool + either *Bacteroides* strain. Gray area indicates confidence interval. Statistical significance was determined by linear model on relative abundance proportions, accounting for strain, time point and sex of mouse, ***p<0.001.
